# Personalized microstructural evaluation using a Mahalanobis-distance based outlier detection strategy on epilepsy patients’ DTI data – Theory, simulations and example cases

**DOI:** 10.1371/journal.pone.0222720

**Published:** 2019-09-23

**Authors:** Gyula Gyebnár, Zoltán Klimaj, László Entz, Dániel Fabó, Gábor Rudas, Péter Barsi, Lajos R. Kozák

**Affiliations:** 1 Magnetic Resonance Research Centre, Semmelweis University, Budapest, Hungary; 2 National Institute of Clinical Neurosciences, Budapest, Hungary; University of Pécs Medical School, HUNGARY

## Abstract

Quantitative MRI methods have recently gained extensive interest and are seeing substantial developments; however, their application in single patient vs control group comparisons is often limited by inherent statistical difficulties. One such application is detecting malformations of cortical development (MCDs) behind drug resistant epilepsies, a task that, especially when based solely on conventional MR images, may represent a serious challenge. We aimed to develop a novel straightforward voxel-wise evaluation method based on the Mahalanobis-distance, combining quantitative MRI data into a multidimensional parameter space and detecting lesion voxels as outliers. Simulations with standard multivariate Gaussian distribution and resampled DTI-eigenvalue data of 45 healthy control subjects determined the optimal critical value, cluster size threshold, and the expectable lesion detection performance through ROC-analyses. To reduce the effect of false positives emanating from registration artefacts and gyrification differences, an automatic classification method was applied, fine-tuned using a leave-one-out strategy based on diffusion and T_1_-weighted data of the controls. DWI processing, including thorough corrections and robust tensor fitting was performed with ExploreDTI, spatial coregistration was achieved with the DARTEL tools of SPM12. Additional to simulations, clusters of outlying diffusion profile, concordant with neuroradiological evaluation and independent calculations with the MAP07 toolbox were identified in 12 cases of a 13 patient example population with various types of MCDs. The multidimensional approach proved sufficiently sensitive in pinpointing regions of abnormal tissue microstructure using DTI data both in simulations and in the heterogeneous example population. Inherent limitations posed by registration artefacts, age-related differences, and the different or mixed pathologies limit the generalization of specificity estimation. Nevertheless, the proposed statistical method may aid the everyday examination of individual subjects, ever so more upon extending the framework with quantitative information from other modalities, e.g. susceptibility mapping, relaxometry, or perfusion.

## Introduction

### Drug resistant epilepsies (DRE)

Drug resistance affects about 20–30% of the epileptic patient population, causing severely impaired quality of life and a difficult to treat situation [1, 2]. Most of the drug resistant cases (~60%) are focal epilepsies, nevertheless there are generalized forms. Malformations of cortical development (MCDs) and long-term epilepsy-associated tumors (LEATs) are among the most frequent etiological factors causing DRE [3–6]. Subtypes of MCDs include focal cortical dysplasia (FCD), polymicrogyria (PMG), heterotopia (HTP), hemimegalencephaly (HME), while subtypes of LEATs include gangliogliomas, and disembryoplastic neuroepithelial tumors (DNTs) [7]. Most of these entities may exhibit variable features on MR images collected with an epilepsy protocol. DRE patients are often candidates for surgical intervention but the probability of postoperative seizure freedom however is remarkably lower in cases lacking any identifiable lesions on conventional MRI [8]. Therefore better visualization of MCDs and LEATs e.g. as shown in [9–15] can be crucial for improving surgical outcomes.

### Diffusion tensor imaging

Diffusion tensor imaging (DTI) is a widely used, albeit rather simplistic, representation of diffusion weighted MRI (DWI, or dMRI) data (applicable with sufficient number of diffusion weighting directions and strength), in the examination of white matter (WM) microstructure [16–18] on the voxel-level, and macrostructure through tractography. Rotationally invariant scalar metrics, derived from the diffusion tensor eigenvalues (λ_1_, λ_2_, λ_3_), such as mean (MD) or radial diffusivity (RD) and fractional anisotropy (FA) [17, 19] are proven to be sensitive to different pathological changes. FA, the normalized standard deviation of the eigenvalues is highly sensitive but aspecific to changes in WM microstructure, and although it has been widely considered as a measure of structural integrity, interpreting its changes, especially in regions with crossing fibers, is a more complex question. Apart from FA the most commonly reported metric is MD, the arithmetic mean of the eigenvalues, which is shown to be proportional to membrane density, while RD (the mean of the second and third eigenvalues) has been shown to increase with changes of the myelin structure (de- or dys-myelination) or increased axonal diameter [20]. The first eigenvalue, often referred to as axial diffusivity (marked either by λ_1,_ L1, AD, or DA), has been reported to decrease with various types of pathologies (e.g. axonal injury [21]) and increase with brain maturation [22].

Typical DTI studies involve group-level comparisons of two or more of these scalar values by the application of parametric tests on regions of interest (ROI, [23]), skeletonized WM (using Tract Based Spatial Statistics—TBSS [24]), or the voxel-level [25, 26]. Nevertheless, selecting which metrics are necessary to be examined has been a debated topic [27], and, as all of them are calculated from the same three eigenvalues and therefore usually exhibit strong correlations, it is arguable whether including all four in any study provides meaningful additional information.

The current study introduces a simple and straightforward statistical evaluation method, which, by examining the three eigenvalues themselves, covers all the scalar information of the diffusion tensor.

When trying to uncover pathological brain regions of individuals, comparing one patient to a group of controls is called for, however the use of conventional parametric tests in such single-subject examinations is always limited, as the fundamental assumption of fixed parameter sets is often violated. Popular approaches to work around this problem in DTI examinations include the use of nonparametric tests [28], or permutation approaches [29], however these methods usually only work with single variables or metrics of interest.

### DTI in the diagnosis of epilepsy

DTI has been proven sensitive to the disrupted tissue microstructure, identified in MCDs. Abnormalities tend to extend beyond the lesions themselves, for example [30] identified decreased FA and increased MD and RD in regions spanning 5-20mm around the nodules in children with periventricular nodular heterotopia. Widespread decrease of FA was also demonstrated in [31] in major WM tracts in both hemispheres (e.g. in the cingulum, forceps minor, anterior thalamic radiation, superior longitudinal fasciculus, uncinate fasciculus, and the inferior fronto-occipital fasciculus) in a group of patients with frontal FCDs, using TBSS.

More sophisticated models such as diffusion kurtosis imaging (DKI; [32]) or the NODDI (neurite orientation dispersion and density imaging, [33]) approach may further improve lesion detection based on diffusion weighted MRI [34–36]. Since DTI is still the most widely used approach, mainly because of its simplicity and clinically feasible acquisition and processing time, we chose to demonstrate our proposed statistical method using DTI data, however, the framework we introduce may be applied to all kinds of voxel-wise variables derived from any meaningful model.

### The multidimensional approach

Multidimensional studies aim to combine information from independent sources in order to raise statistical power; a feat sought after in the neuroimaging literature. Several strategies were employed to implement this combination at different levels of statistical analysis throughout the past two decades, using (and sometimes combining) voxel-wise, surface-based, and ROI-level methods. The performance of this pooling of information has been evaluated on the level of *p*-values [37, 38], *T*-score maps [39], and by using multivariate [40, 41] and logistic regression [42].

The lowest level at which neuroimaging information can be combined is achieved by working with raw data, or derived parameter maps. Such was the approach e.g. in [43] working with voxel-wise MD and volumetry data. More recently, the performance of machine learning based classifiers in the scope of lesion detection was demonstrated with satisfying performance, e.g. on the voxel level in [12], working on T_1_-weighted data using a one-class support vector machine-based classifier and outlier detection approach; or on the vertex-level, working with morphologic and intensity-based metrics in [10] and [44] using surface-based methodology.

As the aforementioned models and studies demonstrated (detailed in 4.1 in the Discussion), multidimensional approaches can increase statistical power by combining the sensitivity profiles of independent modalities, but their usage is often complicated, computationally expensive, and includes arbitrary choices (for example the choice of combining functions by [39] or the choice of weighting factors for multivariate linear regression).

The current study was aimed at developing a more straightforward and easier to use method, based on the Mahalanobis-distance for testing neuroimaging (specifically DTI) data in the context of lesion detection when comparing a single patient to a group of healthy controls.

### The Mahalanobis-distance

#### Definition

The Mahalanobis-distance is a measure of dissimilarity, commonly used in multivariate outlier detection problems [45–47]. Following the original definition by [48], in a *P* dimensional statistical field (constructed from *P* separate variables) the squared distance between an observed distribution with mean ***μ*** *= (μ*_*1*_, *μ*_*2*_, _*…*_ *μ*_*P*_*)* and covariance matrix ***S***, and any point ***X*** *= (X*_*1*_, *X*_*2*_, *…X*_*P*_*)* is expressed in the form:

$$D_{M}^{2}={\left(X-\mu \right)}^{T}\;\underline{\underline{S^{-1}}}\;(X-\mu )$$ (1)

**Fig 1:**
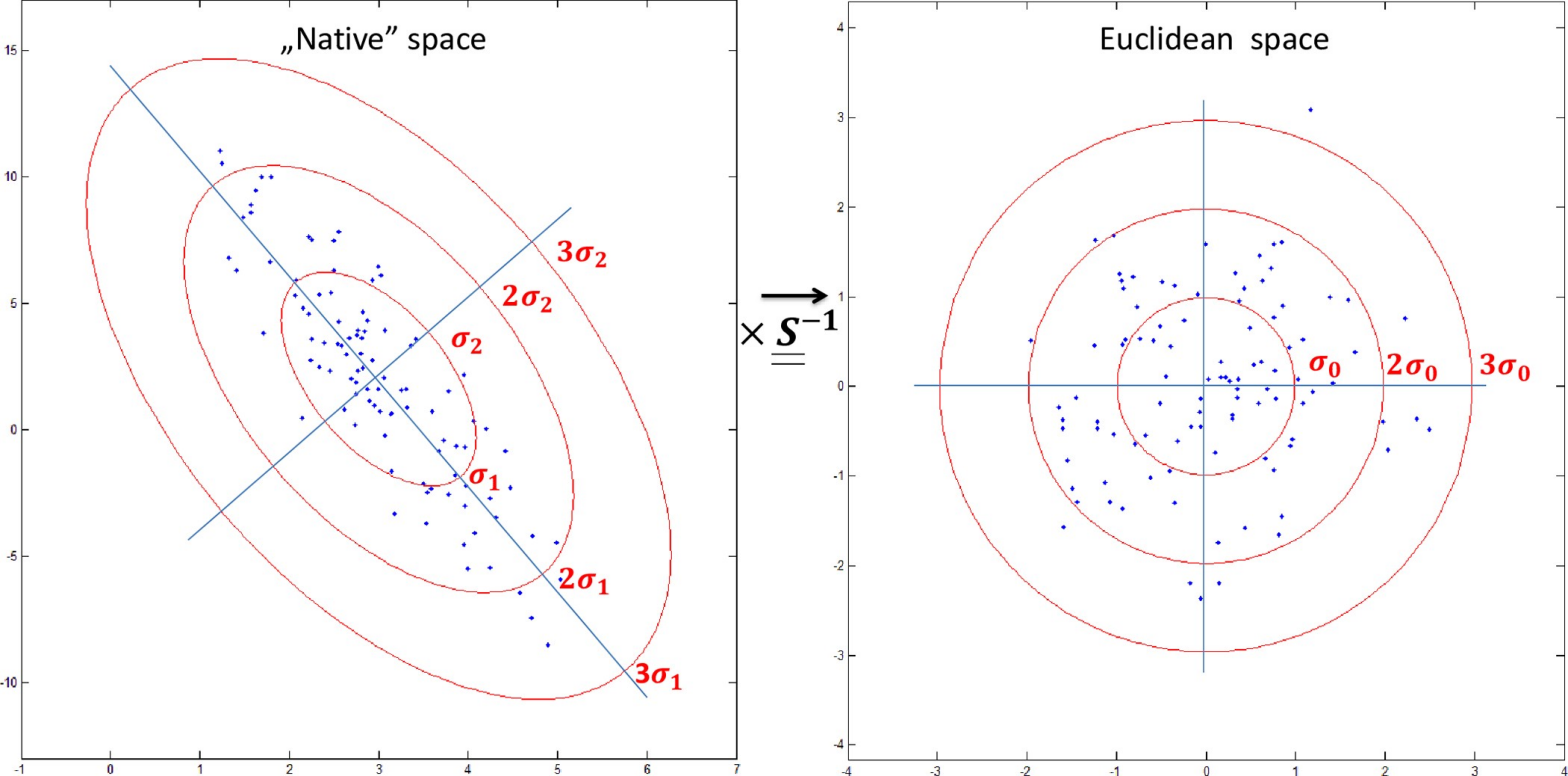
The effect of the multiplication with the inverse of the covariance matrix. The multidimensional distribution is cleared of possible correlations and differences in standard deviation, therefore the distances are effectually calculated in a Euclidean space.

Multiplication with the inverse of the covariance matrix maps the inter-point distances to a standard *L*^*2*^-norm, cleared of any possible correlations and differences in standard deviations *(σ*_*1*_, *σ*_*2*_,*… σ*_*P*_*)* between the dimensions (Fig 1); therefore *D*^2^ values reflect how far a given point is from the underlying multivariate distribution.

This mapping feature is potentially useful in diffusion weighted image processing and the detection of pathological tissue microstructure in the DTI framework, as different tensor eigenvalues are sensitive to different pathologies but they generally exhibit strong correlations [20–22].

By definition, the Mahalanobis-distance is related to Hotelling’s *T*^*2*^ (e.g. used in [43]) with the exception that the latter compares a group of subjects to the reference distribution, by using $\bar{X}$ (the group average of ***X***^***i***^ *= (X*_*1*_, *X*_*2*_, *…X*_*P*_*)* vectors, each corresponding to an individual subject) instead of a single ***X***. Like Hotelling’s *T*^*2*^ is often referred to as the multidimensional equivalent of Fischer’s two-sample *T*-test, one may view the squared Mahalanobis-distance as a multidimensional one-sample *T*-statistic.

The Mahalanobis-distance has been employed in neuroimaging in relation to various disorders and at different levels of information processing: in discrimination between normal tissue types and brain tumor [49]; in ordering the eigenvectors of discriminatory principal component analysis [50], differentiating Schizophrenia patients from controls using whole brain FA; in combining DTI-scalar metrics with T_1_ and T_2_-weighted images in WM-ROIs [51], quantifying brain maturation; in discerning subtypes of mild cognitive impairment [52] based on T_1_, T_2_, and proton density-weighted images; and, more recently, in quantifying the difference between patients with autism spectrum disorder and subjects with normal aging [53], using different sets of DTI scalars from major WM tracts.

**Fig 2:**
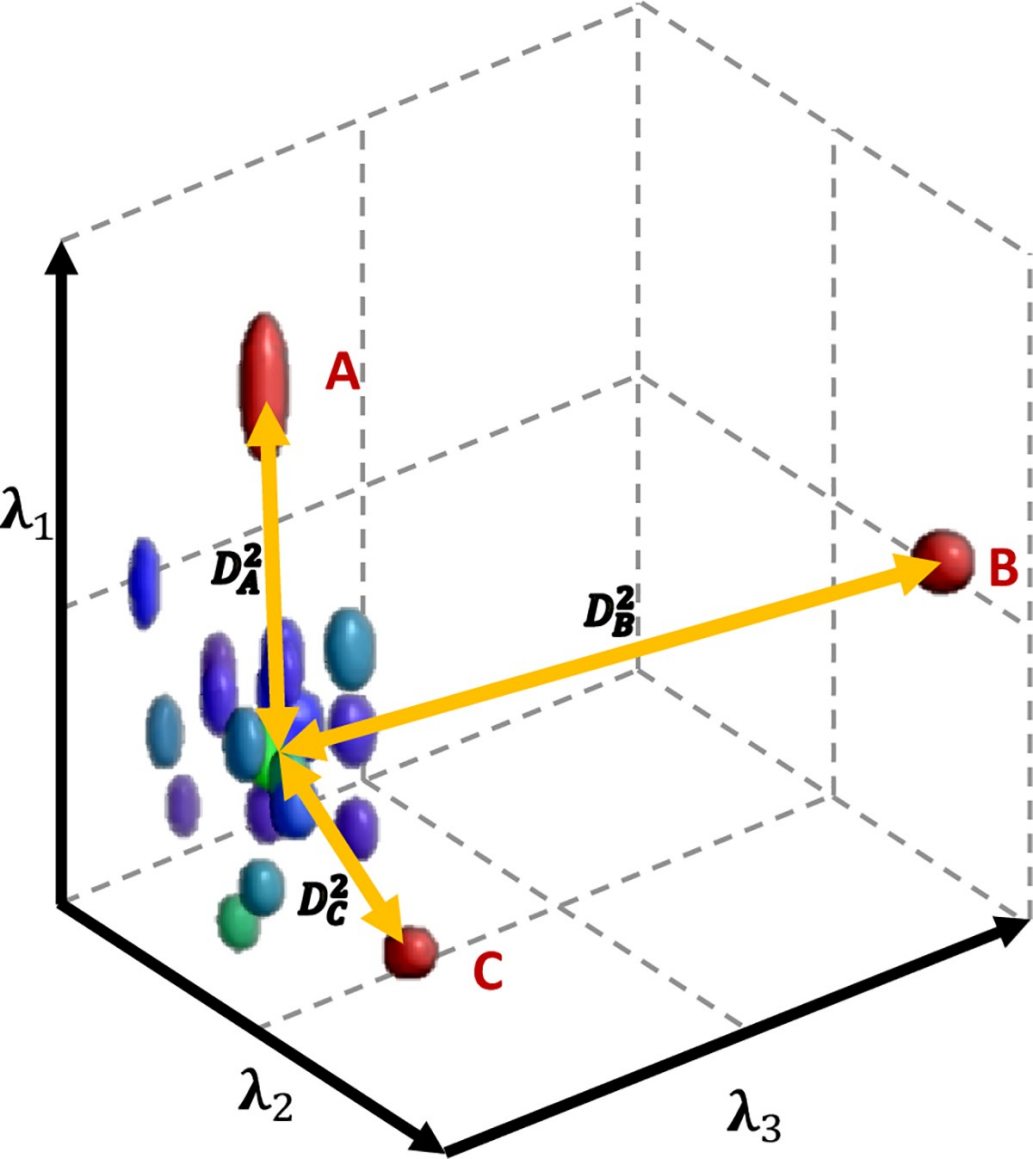
Mahalanobis-distance in the 3D space of DTI eigenvalues. Outlying diffusion profile in a given voxel of a single subject under examination (red) is detectable through the distance (*D*^*2*^) from a group of controls (blue and green) in the three dimensional parameter space of the diffusion tensor eigenvalues. Common alterations of the diffusion profile, such as a higher first eigenvalue (as in the case of point A; usually detected through increased fractional anisotropy in univariate tests); an increase in all three eigenvalues (B; commonly observed as increased mean diffusivity); or an altered diffusion profile with normal-appearing diffusion strength (like in the case of C, when MD equals to the average MD of the controls, but the eigenvalues differ) are all detectable in the multivariate framework with a single test.

In the current study, 3 dimensional distributions were constructed in each voxel from the eigenvalues of the diffusion tensor, and the voxel-wise squared Mahalanobis-distance was calculated using empirical ***μ*** and ***S*** from samples containing one patient and a group of control subjects (Fig 2).

#### Statistical inference based on critical values

Critical values for detecting a single multivariate outlier at a desired level of significance, as shown by [54], can be calculated using Wilks’s criterion [55] with the following formula:

$$D_{crit}^{2}=\frac{p{\left(n-1\right)}^{2}F_{p,n-p-1;\alpha /n}}{n\left(n-p-1+pF_{p,n-p-1;\alpha /n}\right)},$$ (2)

where *p* is the number of dimensions, *n* is the number of observations (subjects) and *F* is the distribution function of the *F* statistics, with the appropriate numerator and denominator degrees of freedom at the desired significance level *α*. By selecting a sufficiently conservative *α*, i.e. one aiming to control the family-wise error rate (FWE) or the false discovery rate (FDR), the problem of multiple comparisons (high number of voxels under examination) may also be addressed. Although the distributions of the diffusion tensor eigenvalues are usually not strictly Gaussian, this generally does not affect the calculation of Mahalanobis-distance significantly, however, it may result in an overestimation of the critical values somewhat reducing sensitivity with the unintendedly more conservative inference. With the analytically derived critical values accounting for sample size, statistical significance is not likely to be affected by the bias described in [56], however, as with conventional statistical approaches, using larger control samples is desirable to increase specificity.

### Spatial registration

For all voxel-level examinations, images under consideration (the DTI eigenvalue maps in our analysis) have to be coregistered, i.e. transformed into a common coordinate system to achieve sufficient spatial concordance. For its high performance, we chose the DARTEL [57] processing pipeline from the SPM12 toolbox (http://www.fil.ion.ucl.ac.uk/spm/) [58]; an approach commonly used in voxel-based morphometry (VBM) studies [59, 60]).

DARTEL creates a ‘template’ image in several iteration steps that is the closest to each individual subject’s anatomy. This way the common coordinate system is tuned for being study-specific, resulting in more efficient handling of macroscopic anatomical differences compared to other widely used approaches, for example those utilizing the MNI152 template space [61] as the target of ‘normalization’.

The popular and widely-used TBSS [24] approach of the FSL software package (https://fsl.fmrib.ox.ac.uk/fsl/fslwiki/) may reach a higher level of spatial correspondence, but it limits the statistics to a supposed WM ‘skeleton’ by projecting voxel-level DTI scalar values to the centerlines of the WM fiber bundles. This spatially informed and highly confined data reduction is suboptimal for detecting lesions that are most prominent in the GM-WM boundary zone; therefore, we opted for extending the scope of the analysis to the whole brain, while aiming to retain high power by using the multidimensional approach, and not using TBSS in our investigation.

As this study’s main objective is aiding the detection of small, hard to find lesions, we chose not to include spatial smoothing (which is generally applied in voxel-level examinations), as it would diminish our method’s performance in such applications.

### Aims

The main aim of the current study was to evaluate the performance of the Mahalanobis-distance as a tool for detecting microstructural abnormalities, by simulations using data from standard multivariate normal distribution (SMVND – *𝓝*_***P***_***(0*,*1)***) and from healthy controls. Based on the simulation results we also aimed to demonstrate the utility of the approach in select cases of patients with MCDs.

## Material and methods

Diffusion and T_1_ weighted MR imaging data of 45 healthy control subjects (25.6 years average age, range: 20–37 years, 17 males) and 13 patients (21 years average age, range: 7–46 years, with two children under 10, 7 adolescents between 14 and 18, 9 males) with MCDs was acquired at 3T (Philips Achieva scanner, Philips Medical Systems, Best, The Netherlands). DW-MR images were collected with a single shot SE-EPI sequence, with diffusion weighting in 32 directions with b = 800 s/mm^2^ and one b = 0 image. In-plane resolution was 2x2 mm (reconstructed to 1.67x1.67 mm with zero filling); whole brain coverage was achieved with 84 (adjusted when necessary), 2 mm thick axial slices and no gap; TR = 9660 ms repetition time, TE = 75.64 ms echo time, and 90° flip angle was used; the total acquisition time was 8:32 minutes. High resolution 3D T_1_ weighted images were also acquired for registration purposes (1mm isotropic voxels), using a standard 3D gradient-echo sequence. 2D fluid attenuated inversion recovery (FLAIR) sequences (0.43x0.43 mm in plane resolution, 3.3 mm thick coronal slices, tilted perpendicular to the hippocampi, TR = 9000 ms, TE = 125 ms, TI = 2800 ms, flip angle = 90°) were also acquired for the visualization of the MCDs.

Patients were selected retrospectively, with several different types of MCDs and other abnormalities: MCD subtypes included polymicrogyria (in two patients) schizencephaly (two patients), subependymal heterotopia (in three patients), FCD (in six patients), hippocampal sclerosis (in four patients), cortical dysgenesis (in three patients) and other, not clearly identifiable malformations (in four patients). Several other types of abnormalities were also identified in the patient group, such as DNT (in one patient, later confirmed by histopathology), ischemic WM lesions (in two patients), a gliotic cyst (in one patient), focal gliosis (in one patient, also confirmed by subsequent histopathology), and malrotation of the hippocampus (in one patient). Diagnoses of MCD subtypes were based on neuroradiology report; the supplementary S1 Table contains detailed information on each lesion and abnormality, along with the results of neuroradiology assessment and lesion detection calculations.

The study was approved by the Scientific and Research Ethics Committee of the Medical Research Council, Budapest, Hungary (ETT TUKEB—20680-2/2012/EKU (368/PI/2012)) for patients and (ETT TUKEB 23609-1/2011-EKU, 23421-1/2015-EKU) for controls; all participants provided written informed consent. Anonymized T_1_-weighted images (facial structures removed using the ‘mri_deface‘ function of Freesurfer - https://surfer.nmr.mgh.harvard.edu/fswiki/mri_deface) and coregistered DTI-eigenvalue maps of the patients and controls are available in the ‘GIN’ public repository under the DOI 10.12751/g-node.80dd9a.

### Data processing

DWI data was preprocessed using the Matlab-based ExploreDTI software package (http://www.exploredti.com/) [62]. Processing steps included the transformation into ExploreDTI’s coordinate system, rigid body transformations for correcting subject motion, and non-rigid transformations for susceptibility-related and EPI-induced distortion-correction, while also rotating the b-matrix (the diffusion directions) accordingly, in order to avoid angular inaccuracies [63]. T_1_-weighted images were used as templates for registration to correct the distortions inherent to the EPI-acquisition method [64]; thereby DW-images were spatially aligned to these T_1_-weighted images. After robust tensor fitting, using the RESTORE (Robust Estimation of Tensors by Outlier Rejection) [65] algorithm, the tensor eigenvalues were calculated and exported for the voxel-level analysis.

As described in subsection ‘Data processing’, we used the DARTEL method with default parameters for the group-level coregistration of the eigenvalue images with the following steps:

The DARTEL template was created from the T_1_-weighted images of the control subjects; patient data was subsequently registered to this common space. As a byproduct of the registration, ‘flow-fields’ describing the transformation between each individual’s native space and the template space were obtained and used to coregister the eigenvalue images. Finally, the DARTEL template was used to generate a brain mask and subsequent calculations were limited to this volume.

This way, the reference distribution of voxel-wise DTI eigenvalues in the common coordinate system (control data) had low observed sample variance, unbiased by patient anatomy, and provided a solid basis for sensitive lesion detection. On the other hand, this subsequent transformation of patient data may have amplified registration artefacts, especially in cases when a patient was highly different from the controls (e.g. when the patient was significantly younger, or had large anatomical abnormalities).

The DARTEL pipeline includes a ‘modulation’ step to account for macroscopic anatomical differences, using the Jacobian of the transformation matrices. As the method was developed to examine cortical thickness and structure, when the transformation includes the merging of voxels, the summation of tissue probability values keeps the information of cortical thickness. However, when working with DTI eigenvalues, this addition (preserving the ‘concentration’) would falsify the original diffusion traits, therefore this ‘modulation’ option was omitted in our processing framework [42]. This processing pipeline contains only 2 interpolation steps. First, in the motion and distortion-correction step, the DWI data is interpolated to the finer resolution of the T_1_-weighted images [66], while the second is performed in the coregistration step of the DARTEL method, to a coarser, 1.5mm isotropic resolution. This is the necessary minimal number of interpolations when each individual’s T_1_-weighted images are used for DWI distortion correction, and statistical inference is made in a common space.

Spatial alignment was assessed by visual inspection and the ‘Check Data Quality’ function of the Computational Anatomy Toolbox (‘CAT12’, an extension to SPM12) [67]. This tool calculates a three dimensional spatial correlation coefficient between images; misaligned data is easily identified by the decreased level of correlation.

The resulting coregistered whole brain tensor eigenvalue images of the healthy subjects were used for three purposes: (a) as data basis for simulations in a ‘bootstrap’ manner, (b) in a leave one out examination to measure the performance of coregistration and its effect on false positives, and (c) as controls when patient data was examined.

#### Independent automatic evaluation of MCDs

As part of our epilepsy post-processing protocol we also used the MAP07 toolbox that performs single subject vs. control group comparisons on volumetric T_1_ data derived 3D feature maps regarding the GM–WM junction (junction map), cortical gyration (extension map), and cortical thickness (thickness map). The resulting *Z*-score maps can be thresholded and/or combined (combined map) in order to pinpoint areas with suspected pathologies [13–15].

We analyzed all our cases using the default processing parameters of the MAP07 toolbox, the feature map comparisons were performed against a generic normal database provided with the software, which consists data of 150 healthy controls scanned on five different MRI systems [14]. The resulting Z-score maps were thresholded at the default *Z > 4* value and then combined and converted to ROIs.

The resulting ROIs were used to signify locations being suspicious of malformation of cortical development in the general neuroradiology workup. They have all been re-evaluated by the neuroradiology expert (PB), and those without underlying pathology were discarded. The MAP07 ROIs deemed relevant, along with the ones manually traced over lesions not being identified by the MAP07 toolbox, were then edited (ZK) to completely cover the respective pathologies, and then served as ground truth lesions in further analysis.

### Mahalanobis-distance related calculations

**Fig 3:**
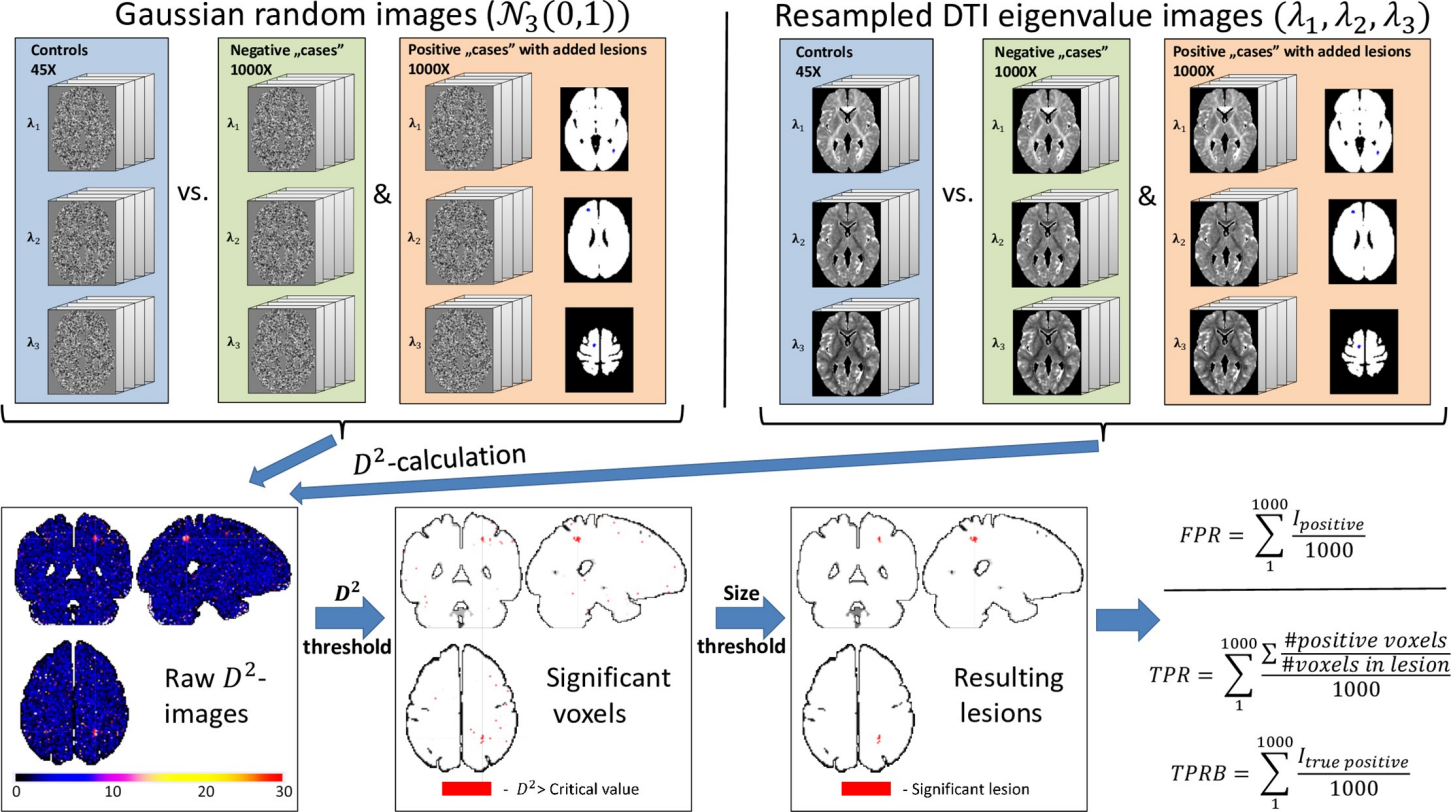
Flow chart demonstrating steps of the simulations. Eigenvalue maps of standard multivariate normal distributions (SMVND–upper half) and random resampling of the real eigenvalue maps of the control subjects (lower half) were used as reference data. True negative and true positive ‘cases’ (with added artificial ‘lesions’, i.e. patches of voxel values of shifted distributions, compared to the background) were generated and the squared voxel-wise Mahalanobis-distance (*D*^*2*^) was calculated in relation to 45 control cases. *D*^*2*^-images were subjected to thresholding using FDR- or FWE-corrected critical values (for multiple comparisons) and cluster size thresholding. False positive rates (FPR) were calculated from true negative ‘cases’, while true positive rates (TPR) and hit rates (TPR-binary, i.e. TPRB) resulted from the positive cases.

We have implemented the calculation of the voxel-wise Mahalanobis-distance (*D*^*2*^) from the DTI eigenvalue maps according to (1), the statistical inference based on critical values determined by (2), and cluster size thresholding, in Matlab scripts and functions (MATLAB 9.2, The MathWorks Inc., Natick, MA United States). Eigenvalue maps are being read in nifty format, transformed to vector format for efficient parallelized calculations, inference is performed voxel-by-voxel, followed by cluster identification, and size thresholding (also see the bottom half of Fig 3). The same framework was used for subsequent calculations, including simulations, leave-one-out examination of controls and patient evaluations.

The analyses were performed as follows:

### Simulations

The performance of the method was evaluated using simulations with two distinct sets of data: (a) Gaussian random images and (b) real diffusion tensor eigenvalue data. Following the fashion of Smith et al. [68], alternative fractional receiver operating characteristics (AFROC) analyses was carried out on both sets.

As the critical values calculated by (2) depend on sample size, and the aim of the simulation study was to provide grounds for later analyses; the same number of control observations (45 subjects) were modelled in both simulations.

In order to evaluate the method’s performance as a lesion detection tool, simulations were carried out with different contrast-to-noise ratios (*CNR—*i.e. effect strengths: difference between mean values of the ‘lesions’ and the ‘background’, measured in units of standard deviation with *σ*_*lesion*_ *= σ*_*background*_), and lesion sizes, with a variable cluster size threshold for controlling the rate of false positives. An overview ‘flow-chart’, describing the steps of the simulations is shown in Fig 3.

A group of 45 ‘control subjects’ were generated following 3D Gaussian distribution, with zero mean and unit standard deviation in all three random variables (*𝓝*_*3*_***(0*,*1)***, SMVND) in each voxel (Leftmost panel in the upper half of Fig 3). The spatial dimensions matched those of the real coregistered DTI data, described in subsection ‘Data processing’.

True positive images were generated, starting from similar random ‘noise’ data and adding simulated ‘lesions’: 3D patches with predefined sizes, randomly generated shape, and voxel values from a distribution with the mean shifted from the background values, according to the predefined CNR. Each true positive image had one ‘lesion’ with a center randomly selected from 25 different locations; coordinates were defined on the template, close to the frontal, temporal, and occipital GM-WM boundary, in view of the second set of simulations with real eigenvalue data. One thousand such positives and another thousand negatives (i.e. just SMVND ‘noise’) were generated to calculate true and false positive rates (Left panel in the upper half of Fig 3).

After the calculation of voxel-wise *D*^*2*^-values, thresholding was performed using critical values calculated to control the Family-Wise Error Rate (i.e. Bonferroni bounds) or the False Discovery Rate (using the Benjamini-Hochberg step-up algorithm on *P*-values calculated by the inverse of (2)). The surviving supra-threshold voxels were subjected to cluster-size thresholding following third-neighbor cluster definition, i.e. 26 neighbors (Middle panel in the upper half of Fig 3).

The resulting binary images were used to calculate the true positive rate (TPR) in positive, and the false positive rate (FPR) in negative cases. Two types of TPR were defined, the first as the ratio of identified positive voxels (i.e. the identified lesion volume ratio, averaged over the pool of positive cases), following the original AFROC TPR as used by [68] (Right panel in the upper half of Fig 3).

As lesion detection is a binary problem (i.e. identifying only a part of the region of pathological tissue is also considered a positive result) any true positive voxel was counted as a hit in the second definition of true positives (TPR Binary—TPRB). False positives were defined similarly, as any positive cluster in a true negative (only noise) image was considered a false hit.

$$TPR=\sum_{1}^{1000}\frac{\sum \frac{\#positive\;voxels}{\#voxels\;in\;lesion}}{1000}$$ (3)

$$TPRB=\sum_{1}^{1000}\frac{I_{true\;positive}}{1000}$$ (4)

$$FPR=\sum_{1}^{1000}\frac{I_{false\;positive}}{1000}$$ (5)

The same sets of simulations were performed for each combination of the controlled parameters with FDR and FWE critical values. TPR, TPRB and FPR values corresponding to each set of controlled parameters were used for the creation of ROC curves and the calculation of ‘area under the curve’ (AUC) values, using the *0–0*.*05* FPR-range, using trapezoids under the curve and the *FPR = 0*.*05* point determined with linear interpolation. AUC values were scaled up to the *[0; 1]* range to compensate for the limited range of interpretation. This constrained FPR-range means that in our simulations, the Family-Wise Error rate is also controlled at the subject level (above the voxel-level FWE or FDR), resulting in thorough correction for multiple comparisons.

Values of the three varied parameters are summarized in Table 1.

**Table 1:** CNR lesion size and cluster size threshold values used in the simulations.

CNR [σ]	1	$2\sqrt{2ln(2)}$^a^	3	$4\sqrt{2ln(2)}$	-	-	-
Lesion size [#voxels]	19	35	50	100	200	-	-
Cluster size threshold [#voxels]	1	2	3	4	5	6	7

^a^ Note that *CNR = 2√(2ln(2))* contrast to noise ratio equals to 1 FWHM distance between the peaks of the distributions.

The desired CNR was calculated by setting the difference between means, in units of standard deviations. In SMVND simulations *σ = 1* was used, while unique values were calculated in each individual ‘lesion’ volume and for each eigenvalue in the second set of simulations with real DTI data.

In an exploratory analysis, additional simulations were performed with smaller effect sizes (down to *CNR = 0*.*1*); however, since the lesion detection performance did not exceed chance level, these results were omitted. Larger cluster size thresholds of *19*, *27* and *50* voxels were also used, but, as no false positives were identified above the size of *4* voxels (*7* voxels in the second set of simulations; see subsection ‘Real Eigenvalue simulations’) these results are not detailed in the present article either.

The second sets of simulations were performed based on the diffusion tensor eigenvalue maps of the control group using bootstrap approach, i.e. *2000* resamples considered as individual ‘subjects’ were generated by random resampling of voxel values from the pool of 45 control subjects (bottom half of Fig 3). Similar to the first set, half of these resamples were designed to be ‘positive’ with added simulated ‘lesions’, while the other half of the resamples was ‘negative’. Finally, the same subsequent TPR, TPRB, and FPR calculations were performed as with the SMVND data, corresponding AUC and optimal threshold vales were obtained.

While the first set of simulations used Gaussian random values in the whole of the brain, the bootstrapping in the second set was performed on the voxel level, thereby these values followed the distribution of tensor eigenvalues in the particular ‘lesion’ volume. Thus the CNR in each artificial ‘lesion’ was determined using a volume-specific *σ* (representing the distribution around the GM-WM boundary), assuming *σ*_*lesion*_ *= σ*_*background*_. Although this may be considered a limitation, as true MCDs are likely to exhibit atypical distribution of tensor eigenvalues, since the statistical decision is made independently in each voxel with no cluster-level inference, this assumption does not affect the detection performance directly.

### Leave-one-out examination of controls

The simulations demonstrated that lesions with sufficiently high effect strengths (CNR) and volumes are detectable using the proposed Mahalanobis-distance based method, with satisfying sensitivity. On the other hand, this high sensitivity makes the approach susceptible to registration artefacts and strong individual variability, resulting in false positive clusters. In order to measure the impact this effect has on patient evaluation, data of the control subjects was also used in a leave-one-out examination, comparing each individual to the remaining 44. Calculation of *D*^*2*^-values, inference with critical values (with FWE or FDR correction), and cluster size thresholding (with the size of 7 voxels) were performed in the same manner as with the simulations. Resulting thresholded *D*^*2*^-maps, indicating regions of significantly outlying diffusion profiles were transformed back to the native space of each patient’s original T_1_-weighted image.

#### Cluster description based on tissue probability maps (TPMs)

**Fig 4:**
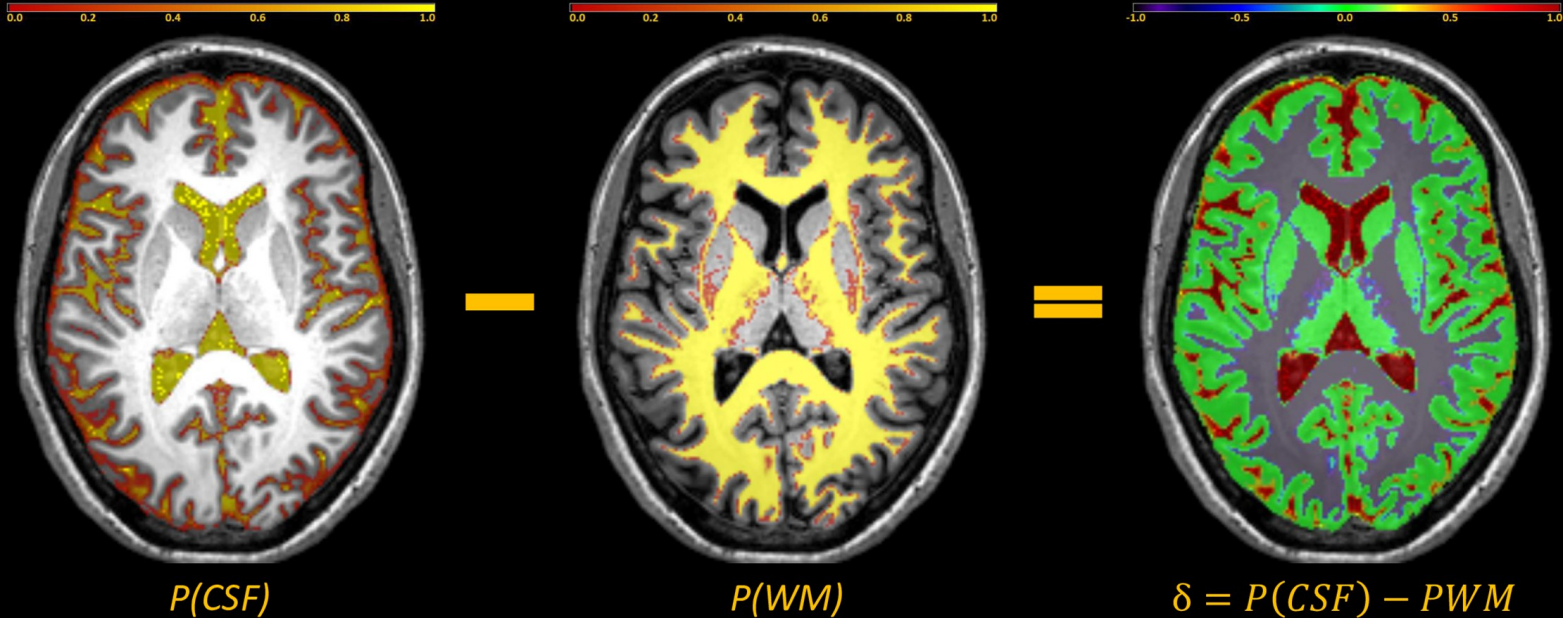
Definition of ‘tissue probability’ used for cluster filtering. δ- values were calculated in each voxel by subtracting the probability of a voxel belonging to the white matter (WM) from the probability of it belonging to the cerebrospinal fluid (CSF), using the tissue probability maps obtained in the initial segmentation of the T_1_-weighted images. The resulting δ-value indicates the voxels’ position along the centrifugal WM-GM-CSF axis.

The results contained several clusters, in many cases obvious false positives, likely resulting from the aforementioned registration inaccuracies and individual variability in gyration patterns. In order to distinguish such false positives and increase the specificity of our method, clusters were subjected to additional post-processing in the following manner:

From each individual’s Tissue Probability Maps (resulting from the initial segmentation step of the DARTEL-pipeline), we defined a new parameter describing voxel position, by subtracting the WM TPM from the cerebrospinal fluid (CSF) TPM: *δ = P(CSF)–P(WM)* (Fig 4). This way a *δ*-value was assigned to each voxel from the *[-1; 1]* range, with positive values indicating voxels closer or belonging to CSF, and negative values indicating voxels closer or belonging to WM.

Registration artefacts around the brain surface would mainly contain voxels with positive values (*δ > 0*; meaning that the majority of voxels are from the CSF). On the other hand, MCDs under consideration typically occur around the GM-WM boundary, thereby true clusters would contain negative values close to zero (*δ ≲ 0*), the distribution of the *δ* -values in any given cluster could be used as an indicator of cluster position along the centrifugal WM-GM-CSF axis.

In the second step, clusters with more than half of the voxels with *δ > 0*.*1* were eliminated from the analysis. This cutoff, signaling lesions with the majority of voxels from the CSF, was determined based on the results of leave-one-out examination of controls.

### Representative cases

As described in subsection ‘Data processing’, DTI eigenvalue maps of patients with MCDs were registered to the DARTEL-template created from control data. *D*^*2*^-calculation and thresholding using FWE-corrected critical values (see the corresponding subsection ‘Real data examinations’ for the reasoning behind using the more conservative correction), cluster size thresholding (again with 7 voxels threshold size), and the *δ*-value-based post-processing of the clusters were performed in the same manner as described above.

Results were qualitatively evaluated by comparing the anatomical images and *D*^*2*^ ‘heatmaps’ along with the *Z*-scored junction maps, resulting from independent calculations by the MAP07 toolbox [13]. Clusters of outlying diffusion profile, remaining after the thresholding and artefact removal steps were considered true positive, when good spatial concurrence with the underlying pathology (as observed on anatomical scans) and the reviewed and corrected results of the MAP07 toolbox was ascertained.

An additional step included the calculation of the clusters’ centers of mass (using the *D*^*2*^-values as weights), and their (physical) distance from lesion masks created manually, based on the neuroradiology reports and aided by independent MCD detection using the MAP07 toolbox (ZK, and LRK) [15] and reviewed by an expert neuroradiologist (PB).

## Results

The FWE-corrected, *D*^*2*^ critical value, calculated using (2) (with *n = 46*, *p = 3*, and $\alpha_{FWE}=\frac{0.05}{\#voxels}=\frac{0.05}{3.4054\times 10^{5}}=1.4683\times 10^{-7}$) was **27.8324**.

As FDR correction uses the *p*-values of each statistical test and determines the critical *p* for a given set (the tests in each voxel, in our case), the FDR corrected *D*^*2*^ critical values were unique for each patient image, typically in the range between **19** and **21**.

**Fig 5:**
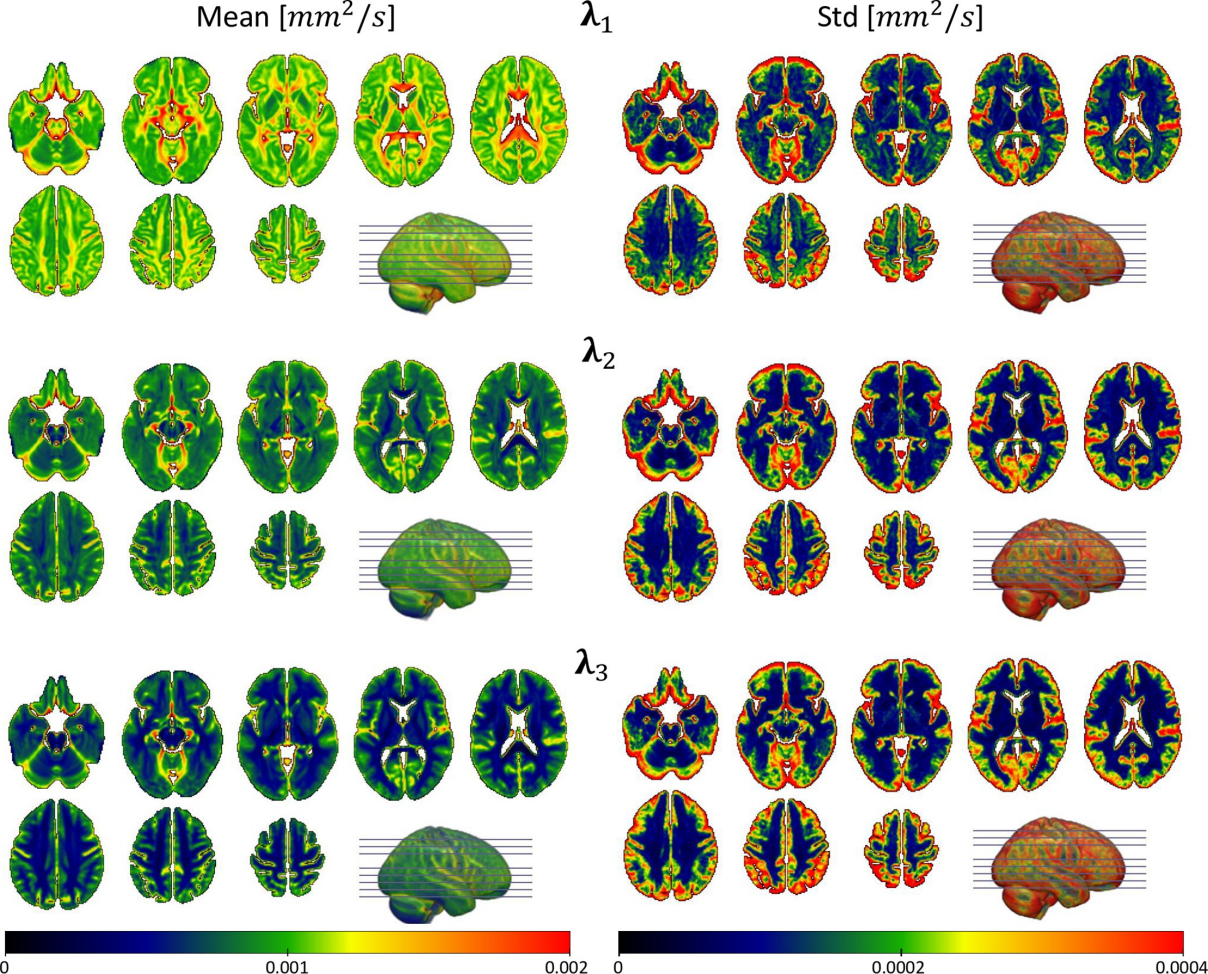
Spatial distribution of the sample-wise mean and standard deviation of the coregistered eigenvalue maps from the controls. Mean values (left column) and standard deviations (right column) are presented on the same respective scales for the three diffusion tensor eigenvalues in units of mm^2^/s.

Group-wise average values and standard deviation for the whole grey and white matter of the coregistered eigenvalue maps from the controls are included in Table 2 while their spatial distributions are presented in Fig 5.

**Table 2:** Mean values and standard deviations of the tensor eigenvalues.

	Grey Matter		White Matter	
	Mean	Std	Mean	Std
λ_1_	1,1295E-03	2,3623E-04	1,2117E-03	2,5012E-04
λ_2_	9,3691E-04	2,1694E-04	8,0431E-04	1,6876E-04
λ_3_	8,1112E-04	2,1290E-04	6,0377E-04	1,7571E-04

Means and standard deviations (Std) of the DTI eigenvalues, averaged over the control sample, in the whole grey and white matter, presented in units of mm^2^/s.

During manual revision of the results of independent lesion detection with the MAP07 toolbox, only 11 abnormalities were identified in the example cases, thereby the remaining lesion masks were entirely hand drawn.

### SMVND simulations

#### False positives

In the simulations with SMVND data, false positives were identified in all cases when no cluster size thresholding was employed, with both FWE and FDR corrected critical values. On the contrary, no false positives were identified with thresholds larger than 4 voxels, meaning that for simulated lesions with voxel values from standard Gaussian distribution, and sizes that are reasonable to assume any true malformation would have, the method had 100% specificity.

#### True positive rates and hit rates

**Fig 6:**
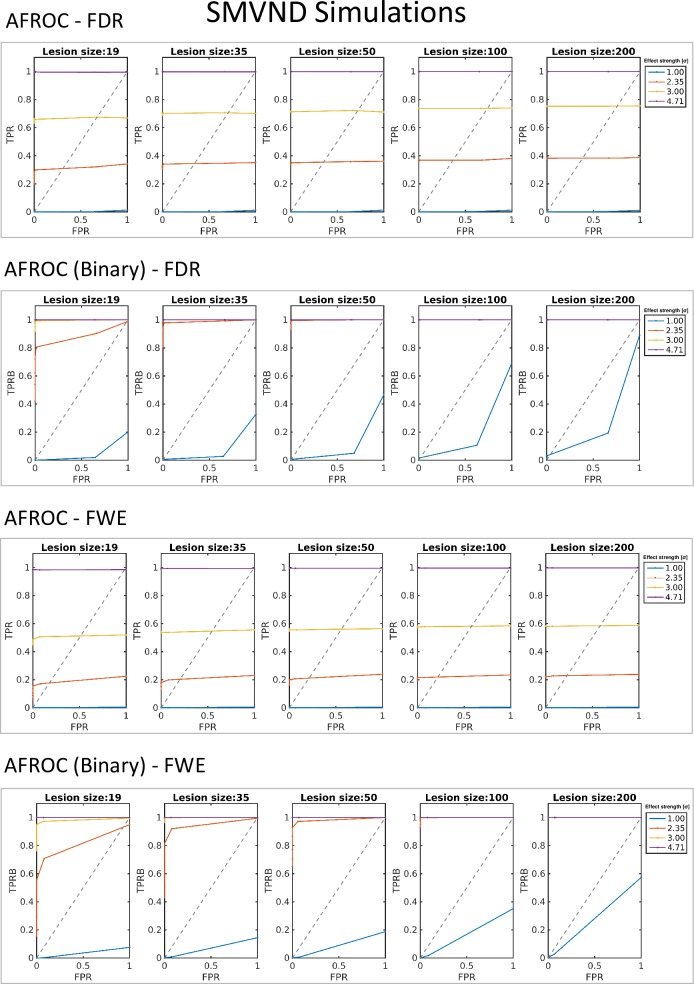
Alternative fractional receiver operating characteristics (AFROC) curves of the simulations with standard multivariate normally distributed (SMVND) data. Results with both FWE- and FDR-corrected critical values, following both definitions of true positives (fraction of positive voxels–TPR–and hit rates–TPRB), with all different values for simulated lesion size and effect strength (contrast to noise ratio) are presented.

AUC values, calculated for each lesion size-CNR parameter pair, with both FWE and FDR corrected critical values are summarized in Table 3 for both definitions of true positives (AFROC curves can be seen in Fig 6). As expected, with increasing CNR and lesion sizes, both the TPR and the TPRB (hit rate) increased.

**Table 3:** AUC value results of the simulations with SMVND data.

SMVND										
FDR										
Lesion size [vox] →	19		35		50		100		200	
CNR ↓	AUC	AUC Binary	AUC	AUC Binary	AUC	AUC Binary	AUC	AUC Binary	AUC	AUC Binary
2σ	0,000	0,000	0,000	0,004	0,000	0,003	0,000	0,005	0,001	0,011
*1 FWHM*	0,296	0,593	0,339	0,840	0,349	0,941	0,368	0,998	0,382	1,000
3σ	0,660	0,953	0,702	0,998	0,713	0,999	0,736	1,000	0,752	1,000
*2 FWHM*	0,996	1,000	0,998	1,000	0,999	1,000	0,999	1,000	0,999	1,000
FWE										
Lesion size [vox]c →	19		35		50		100		200	
CNR ↓	AUC	AUC Binary	AUC	AUC Binary	AUC	AUC Binary	AUC	AUC Binary	AUC	AUC Binary
2σ	0,000	0,000	0,000	0,004	0,000	0,003	0,000	0,005	0,000	0,011
*1 FWHM*	0,158	0,593	0,185	0,840	0,201	0,941	0,216	0,998	0,222	1,000
3σ	0,493	0,953	0,536	0,998	0,555	0,999	0,576	1,000	0,580	1,000
*2 FWHM*	0,984	1,000	0,991	1,000	0,994	1,000	0,995	1,000	0,996	1,000

Area under the curve (AUC) values resulting from the alternative fractional receiver operating characteristics curves (AFROC) of simulations with standard multivariate normal distribution (SMVND) data, FDR and FWE corrected critical values, and following both the fractional and binary definition of true positive rate (TPR); calculated from the *[0; 0*.*05]* false positive rate (FPR) range.

In the *[0; 0*.*05]* FPR interval, the chance level (0.5) was exceeded in lesion detection with all lesion sizes and CNR above 1 FWHM using either FDR or FWE-corrected critical values. More than half of the lesion voxels were identified with *CNR>3σ*, with all lesion sizes and critical values (except for the smallest lesions and FWE correction, were the AUC was *0*.*493*).

### Real eigenvalue simulations

#### False positives

Simulations based on real DTI eigenvalue data resulted in similar behavior of false positives: every case showed false positive clusters with a minimum size of one or two voxels, but with cluster size thresholds of 6 (with FWE-correction) or 7 (with FDR-correction) voxels, FPR decreased to *0*.*1–0*.*3%* (i.e. *1–3* false positives per sets of 1000 simulations).

#### True positive rates and hit rates

**Fig 7:**
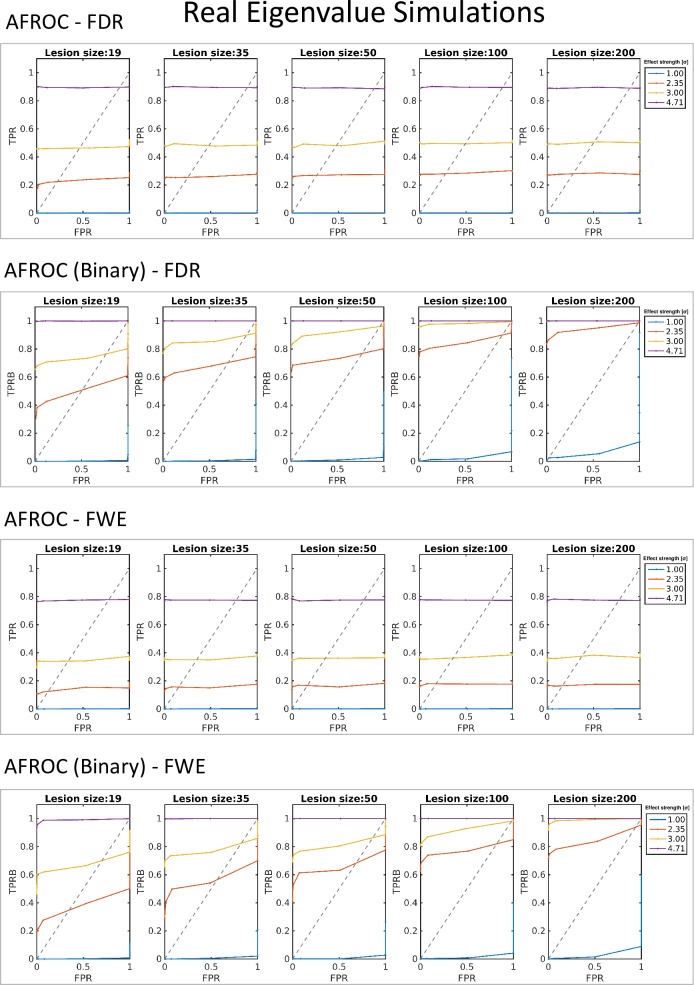
Alternative fractional receiver operating characteristics (AFROC) curves corresponding to the simulations based on real diffusion tensor eigenvalue data. Results with both FWE- and FDR-corrected critical values, following both definitions of true positives (fraction of positive voxels–TPR–and hit rates–TPRB), with all different values for simulated lesion size and effect strength (contrast to noise ratio) are presented.

The resulting AUC values are summarized in Table 4; AFROC curves are presented in Fig 7.

**Table 4:** AUC value results of the simulations with SMVND data.

Real Eigenvalues										
FDR										
Lesion size [vox] →	19		35		50		100		200	
CNR ↓	AUC	AUC Binary	AUC	AUC Binary	AUC	AUC Binary	AUC	AUC Binary	AUC	AUC Binary
2 σ	0,000	0,000	0,000	0,000	0,000	0,001	0,000	0,002	0,001	0,018
*1 FWHM*	0,161	0,299	0,228	0,533	0,229	0,596	0,248	0,702	0,272	0,863
3 σ	0,430	0,637	0,450	0,756	0,414	0,740	0,456	0,884	0,463	0,939
*2 FWHM*	0,773	0,858	0,843	0,940	0,859	0,960	0,822	0,920	0,818	0,920
FWE										
Lesion size [vox] →	19		35		50		100		200	
CNR ↓	AUC	AUC Binary	AUC	AUC Binary	AUC	AUC Binary	AUC	AUC Binary	AUC	AUC Binary
2σ	0,000	0,000	0,000	0,000	0,000	0,001	0,000	0,001	0,000	0,001
*1 FWHM*	0,108	0,221	0,141	0,403	0,160	0,544	0,168	0,696	0,166	0,752
3σ	0,337	0,598	0,353	0,712	0,352	0,745	0,356	0,835	0,353	0,946
*2 FWHM*	0,751	0,947	0,776	0,998	0,778	0,999	0,777	1,000	0,760	0,980

Area under the curve (AUC) values resulting from the alternative fractional receiver operating characteristics curves (AFROC) of real eigenvalue simulations with FDR and FWE corrected critical values and following both the fractional and binary definition of true positive rate (TPR); calculated from the *[0; 0*.*05]* false positive rate (FPR) range

Chance level lesion identification performance (Binary *AUC>0*.*5*) was exceeded at *CNR = 1FWHM* in cases of lesions larger than 19 voxels using FDR-corrected critical values, and in cases of lesions larger than 35 voxels with FWE-corrected critical values; however, performance in lesion voxel identification only exceeded the chance level at *CNR = 2FWHM*, achieving *77*.*3–85*.*9%* AUC with FDR-corrected, and *75*.*1–77*.*8%* AUC with FWE corrected critical values.

Based on these simulation results, it is reasonable to expect that in patient examinations using the diffusion tensor eigenvalues, the proposed method can identify regions exhibiting abnormal diffusion profile, while sufficiently eliminating false positives. Using FDR-corrected critical values and a seven-voxel cluster size threshold should be a suitable choice when searching for small, well-defined lesions.

### Real data examinations

#### Leave-one-out analysis of controls

**Fig 8:**
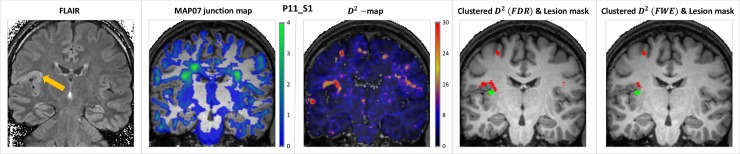
Comparison of the results with FDR and FWE corrected critical values. Thresholded and clustered *D*^*2*^-results overlaid on the T_1_-weighted image of a 27 y.o. male patient with polymicrogyria in the basal region of the left inferior frontal gyrus (see panel D of Fig 8, as well.) Coronal slices presented in neurological orientation, i.e. left side is on the left, slices of the 2D FLAIR image were angulated perpendicular to the hippocampi.

The FDR-corrected critical values resulted in an average of *21*.*11 (5‒55)* clusters/subject, while the more conservative FWE-correction yielded *4*.*93 (0‒13)* clusters in average, *1*.*79 (0–5)* of those being in the WM. Based on this result, combined with the observation that the true positive clusters in subsequent patient examinations were also present with the more conservative approach (Fig 8), we decided to only use critical values aimed to control the FWE for patient examinations, decreasing the influence of inherent variability and/or coregistration inaccuracy.

After removing clusters based on the *δ*-values (those with more than half of the voxels with *δ > 0*.*1*), the number of remaining clusters decreased to an average of *2*.*79 (0‒7)* with an average size of *16*.*21* voxels *(7‒167)*, meaning, that most of those resulting from insufficient coregistration or normal differences in gyration patterns (mainly located in the CSF) were filtered out. Examples of the resulting few minimal cluster-masks overlaid on each control subject’s T_1_-weighted images are shown on S1 Fig.

#### Patient examination

**Fig 9:**
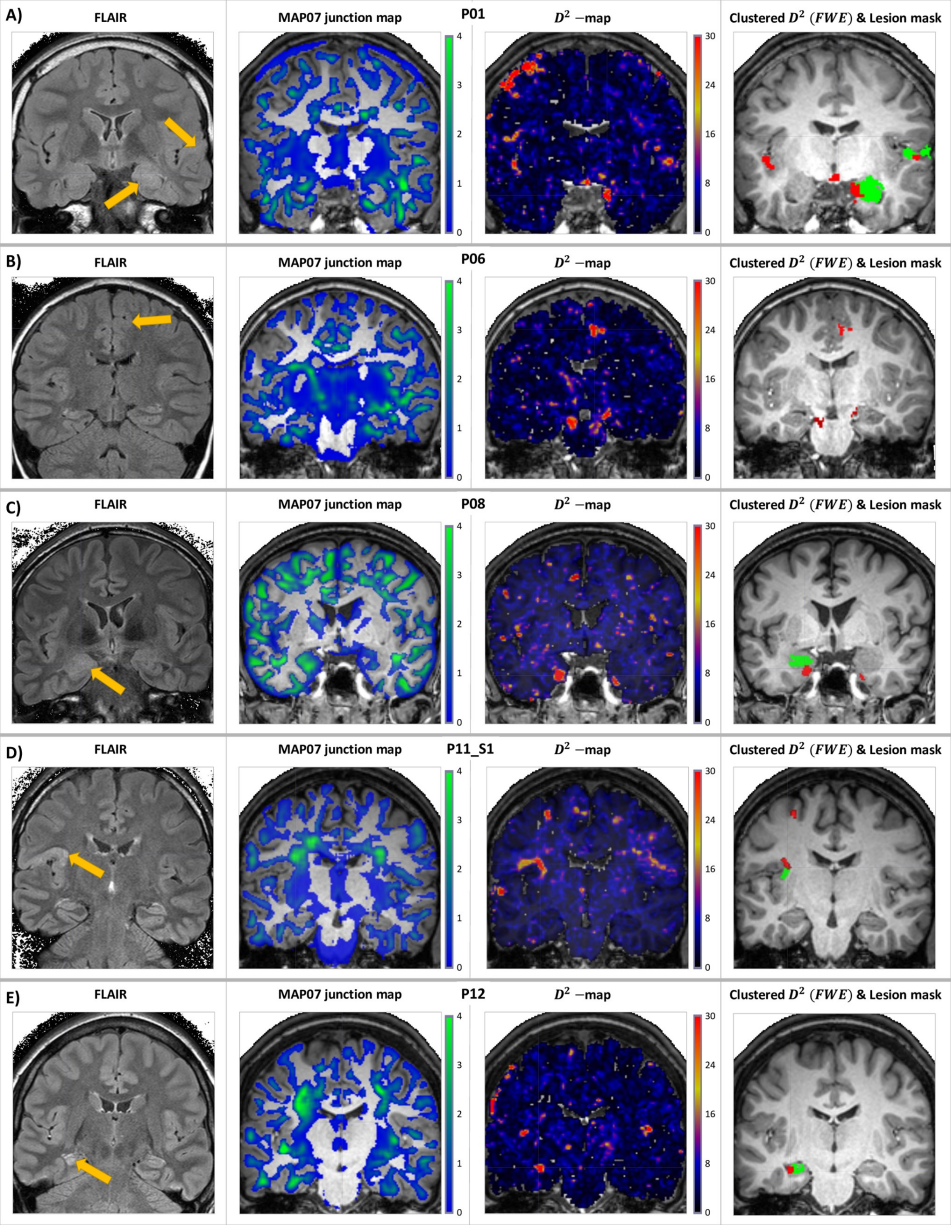
Example results in select cases of MCDs. Coronal 2D FLAIR images, and MAP07 junction maps, raw, and final, clustered *D*^*2*^-images (red) and lesion masks (green) overlaid on T_1_-weighted images in select cases: presumed right superior temporal FCD or PMG and hippocampal sclerosis (panel A); cortical dysplasia and presumed PMG in the right medio-frontal part of the cingular gyrus (panel B); dysgenesis and partial sclerosis of the left hippocampus (panel C); presumed PMG or FCD in the basal region of the left inferior frontal gyrus and the posterior pat of the insula (panel D); and left hippocampal sclerosis (panel E). Coronal slices presented in neurological orientation, i.e. left side is on the left, coronal slices of the 2D FLAIR images were angulated perpendicular to the hippocampi.

After applying the previously detailed processing steps to the 16 *D*^*2*^-images of the 13 patients, on average *59*.*4 (35–90)* clusters per subject were identified with an average size of *31*.*4 (7–680)* voxels (after removing 6 larger clusters emanating from missing cerebellar slices). The majority of these clusters were obvious artefacts, identifiable by their shape and location (e.g. in the occipital lobes, close to and following the GM-CSF boundary, independent of the underlying gyral and sulcal pattern), see Discussion. Examples of resulting clustered *D*^*2*^-images are shown on the rightmost panels of Fig 9 and S3 Fig, along with coronal FLAIR images, MAP07 junction maps and the raw *D*^*2*^-images overlaid on each subject’s T_1_-weighted image. Regions with outlying diffusion properties, corresponding to 22 (out of the 23) MCDs and other abnormalities were identified in the patient group, in good spatial concurrence with the neuroradiological evaluation and the lesion masks. The remaining, 23^rd^, an FCD-type malformation only resulted in two supra-threshold voxels, subceeding cluster size threshold; it was only identified when using the less conservative, FDR-corrected critical values. The (physical) distances between centers of masses of the resulting *D*^*2*^-clusters and the lesion masks are summarized in Table 5.

**Table 5:** Positive clusters in select cases of MCDs.

Code	P01	P02	P03	P04	P05 S1	P05 S2	P05 S3	P06	P07	P08	P09	P10	P11 S1	P11 S2	P12	P13
Number of positive clusters	1	3	1	1	3	3	7	2	1	1	2	3	1	1	2	4
Average distance (min-max) [mm]	12.2	18.8 (12.9–25.7)	6.5	13.6	13.6 (9.44–17.2)	10.4 (5.1–17.1)	19.0 (9.5–29.2)	19.6 (3.7–35.4)	13.5	11.9	12.1 (6.7–17.6)	12.1 (3.5–9.9)	7.7	7.7	4.3 (2.3–6.3)	17.6 (10.2–24.1)

Number of positive *D*^*2*^-clusters, and the average, minimum, and maximum distances [mm] between their and the lesion masks centers of mass

## Discussion

### Regarding multidimensional approaches

When selecting a multidimensional approach to combine different modalities, the research question determines the level at which information is pooled, from group-level, through individuals, down to voxel-based methods. Examining more general processes, like response to stimulation in functional MRI (fMRI) calls for ‘cohort level’ statistical methods, like combining *p*-value maps with pooling approaches in [38], or using the conjunction method by [37], testing a simultaneous null hypothesis.

Higher level information pooling has also been proven efficient in examining systemic disorders of the CNS, e.g. in Alzheimer’s disease in [39], combining *T*-score maps from univariate parametric tests on GM density and perfusion data; or in amyotrophic lateral sclerosis, with multivariate linear regression on spectroscopy findings of different metabolites [41]. The superiority of multivariate models compared to combined univariate models was demonstrated by [40] in examining simultaneous changes in FA, cortical thickness, and perfusion also in Alzheimer’s disease, and logistic regression was shown to improve categorization of patients with different subtypes of mild cognitive impairment in [42] by combining ROI-level DTI, volumetry, and cortical thickness data at the subject level.

On the other hand, when searching for unique abnormalities (like injuries or MCDs) in individuals, inference is made below the subject level: combining data from independent modalities into multivariate distributions and performing statistical evaluation in this high dimensional space enables the pooling of information on the lowest level, only preceded by necessary spatial coregistration. An example for the resulting increased sensitivity was [43] where the combination of voxel-wise MD and volumetry data (using Hotelling’s *T*^*2*^ test, a two-sample equivalent of the Mahalanobis-distance) outlined the effects of traumatic brain injury (TBI), even in cases where none of the individual modalities yielded significant results.

More recent studies also demonstrated the utility of machine-learning based approaches for epileptic lesion detection. Surface-based methodology formed the basis of [10] and [44] using morphologic and intensity-based features (such as cortical thickness, sulcal depth, curvature of the surface, and gradient of intensity; all calculated from T_1_ or T_2_-weighted images on the vertex-level), with similar performance as our approach. In [10] higher specificity was achieved in detecting FCD type lesions.

Similarly to the present study, outlier-detection approach was used in [12], identifying epilepsy-related malformations, using a voxel-based, one-class support vector machine classifier. By working on feature maps computed from T_1_-weighted data, comparable sensitivity and less false positives were achieved than with our approach, partially due to a far more conservative cluster size threshold (82 voxels, compared to 7 in our work).

Such machine-learning based methods are expected to lead the analysis of multidimensional neuroimaging data; however, our study draws merit from several advantages. The straightforward and easy-to-use application of the multidimensional statistics with moderate computation times (only a few seconds per subject on a commercial PC, after preprocessing and registration) aided the accessibility of the method, while the use of DTI data opened the scope of research to disruptions in tissue microstructure.

### On information sources and dimensionality considerations

Theoretically, there is no limitation to the number of examined dimensions in the multivariate distribution examined with the Mahalanobis-distance (as long as the number of subjects exceeds the number of dimensions). Therefore, in order to circumvent the limitations of the diffusion tensor representation, any diffusion processing model (e.g. diffusion kurtosis imaging [32, 34], spherical deconvolution [69, 70], etc.), or even raw diffusion weighted data could be evaluated in the same straightforward manner.

On the other hand, since *L*^*2*^-type distance metrics tend to show decreasing performance with higher number of dimensions [71], known as the effect of distance concentration, and, as [56] demonstrated, the calculation of the Mahalanobis-distance may induce a bias, dependent on sample size, that becomes substantial with higher *(P>10)* number of dimensions, simply pooling together every available source of information would not necessarily increase statistical power. Other types of distance metrics, particularly an *L*^*P*^-norm should be a viable choice in such higher dimensional examinations [72], however, this was out of the scope of the current study.

Another intriguing possibility for MRI-based lesion detection using the Mahalanobis-distance is including voxel-level data from other modalities, such as T_1_ or T_2_-weighted images, tissue probability maps, MRI or PET-based perfusion measurements, etc., as long as proper spatial coregistration is achievable [43]. Since data in any given dimension is rescaled and cleared of correlations, any meaningful modality could be incorporated to the analysis framework, also including more complex measures from related processing pipelines, such as cortical parcellation, volumetry or morphometry results [10, 42, 44], once again, with distance concentration kept in mind.

Feature selection based on the analysis of meaningful components in such an extended parameter space may be the aim of future investigations. Quantitative imaging, a feat currently under intensive research [73], may also benefit from the use of multidimensional distance-metrics in statistical evaluation.

### Regarding simulation results

Simulations with standard multivariate Gaussian data were used to demonstrate the numerical stability and lesion detection performance of the calculations based on the Mahalanobis-distance. AUC values calculated from the *[0; 0*.*05]* FPR range indicated that above 1 FWHM mean difference the method is sufficiently sensitive to even the smallest artificial lesions, using critical values aimed to control either the family-wise error rate or the rate of false discoveries. This is the level of sensitivity typically aimed for in image processing or spectroscopy as the resolution (the minimal distance between two peaks, required to separate them) is generally defined as 1 FWHM.

The use of eigenvalue maps of the control population yielded similar performance: for effect strengths and lesion sizes expected in MCDs (i.e. 50 voxels, corresponding to *168*.*75mm*^*3*^ volume, around 5-7mm in diameter, [12]) the proposed method effectively identifies regions of abnormal diffusion profile. This performance is on pair with that e.g. [68] achieved in simulations introducing the threshold-free cluster enhancement (TFCE) method. Although the distribution of the tensor eigenvalues was not exactly Gaussian, this only resulted in a small reduction in observed sensitivity, which did not cause any substantial reduction in lesion detection performance.

False positives were completely eliminated in simulations on Gaussian random data, with cluster size thresholds of 4 voxels, but a 7 voxel threshold was needed to reduce the FPR to *0*.*1–0*.*3%* in simulations based on the resampling of real eigenvalue maps. (With both FWE and FDR corrected critical values.) Additional exploratory analysis (not presented in this article) using larger thresholds (19, 27, and 50 voxels) confirmed the complete elimination of false positives, at the cost of reduced sensitivity (reduced true positive rates) to smaller lesions.

Based on these findings we concluded that a cluster size threshold of 7 voxels (i.e. one voxel and its nearest neighbors) should be an optimal choice for lesion detection, when no spatial smoothing is performed on the diffusion tensor eigenvalue images. Only this value was used in the following examinations of healthy controls and patients with MCDs.

Outlier values emanating from measurement errors or numerical instability usually affect single voxels, thereby false positives of such origin could effectively be eliminated with the cluster size threshold of seven voxels. For applications with statistical inference performed on images with substantially different resolution from that of the acquisition (e.g. if the eigenvalue images are resampled to a much smaller voxel size during the processing), an adjusted cluster size threshold (covering roughly the same volume as 7 voxels with the acquisition voxel size) should achieve similar robustness to such effects. Similar consideration should go for applications where spatial smoothing is performed at some level during data processing.

### Regarding the leave-one-out examination of controls

Use of the more conservative FWE-corrected critical *D*^*2*^-values and the TPM-based cluster-evaluation method (*δ*-values) limited the number of false positives to an acceptable level. Examination of the control subjects demonstrated that even with the high performance DARTEL-coregistration, clusters of voxels with outlying diffusion profile tend to emerge in (supposedly true negative) control subjects. Most of these clusters proved to be indeed artefactual, being outside the brain parenchyma, however, in average *1*.*79* clusters/ subject were identified in the WM as well. At this level there is no discrimination between clusters emanating from individual anatomical variability and insufficient registration or noise; this problem is usually addressed (reduced) by spatial smoothing in most voxel-level studies [74], which we omitted to retain sensitivity for smaller lesions.

### Regarding patient examinations

Apart from one case, all of the MCDs and other abnormalities in all patients were identified on the processed *D*^*2*^-images, demonstrating the sensitivity of our diffusion-tensor based approach for detecting minute structural abnormalities. The remaining one FCD-type malformation was identifiable only in results obtained with the more liberal, FDR-corrected critical values. This observation demonstrates that the conservative approach with strict critical values can result in false negatives, thereby decreased sensitivity, in brain regions where the DTI eigenvalues in the control group showed higher sample variance.

Raw *D*^*2*^-‘heat maps’, MAP07 ‘junction maps’, and the final *D*^*2*^-clusters were reviewed with an expert neuroradiologist (PB) and compared to the ground truth lesion masks. As the MCDs under consideration are mainly localized around the WM-GM boundary (MAP07 also compares voxels from T_1_ images focused on this compartment) and DTI is expected to be more sensitive in the WM, in most cases, *D*^*2*^-clusters did only partially overlap with the lesion masks; hence concurrence was ascertained by spatial adjacency. The physical distance between the lesion masks and the *D*^*2*^-clusters’ centers of masses was also recorded; in clusters deemed positive, the average distance was *12*.*07mm*, in agreement with results from literature [32].

Patient data was registered to the DARTEL-template, created from only the controls. With this approach, the template was well defined with relatively low sample variance in diffusion tensor eigenvalue distributions, nevertheless artefactual clusters were commonly observed, but they were present mainly in the CSF or around the GM-CSF boundary. The *δ*-value-based method, evaluating clusters based on tissue probability maps was efficient in filtering out the more evident ones, however, several cases showed obvious artefacts identifiable by their shape and location (e.g. in the occipital lobes, close to and following the GM-CSF boundary, independent of the underlying gyral and sulcal pattern) escaping elimination. Additional automatic classification of artefactual clusters based on spatial distribution properties similar to those implemented in SOCK [75] and FIX [76] would further aid the evaluation of results. Since the focus of the present study was on the statistical approach for examining tissue microstructure and the surviving artefactual clusters were easily discernable among the results, thus did not severely obstruct patient evaluation, we chose to favor generality and did not penalize the examined volume any further. Utilizing any or a combination of the above mentioned filtering or labeling approaches would possibly increase lesion detection specificity, but the detailed evaluation of cluster features is clearly outside the scope of this paper, and may be investigated in future studies.

In most patients, smaller clusters (typically under 50 voxels) further away from the actual lesions were also identified in the WM. Apart from the ones in the terminal WM, found in several of the adolescent patients, likely reflecting age-related differences in myelination; based on previous studies [30, 31], such extended WM-abnormalities are to be expected in epileptic patients [3]: they most likely reflect either the underlying pathological networks or compensatory effects or elicited by them [77]. Exploratory analysis of DTI tractography data in select cases demonstrated that most of these additional WM clusters are indeed located in or close to the fiber pathways passing through the primary lesion volumes (S2 Fig).

Such clusters suggest that microstructural changes reflected in the DTI data is not specific to the malformations themselves, but also to the disruptions, the actual MCDs inflict on the corresponding WM pathways. In qualitative evaluation, they may be of clinical importance shedding light on the extent and/or organization of the epileptic networks themselves. Nevertheless, including other sources of information (e.g. relaxometry, susceptibility, perfusion, or morphometry measurements) in the proposed multidimensional statistical framework is likely to improve lesion detection specificity. Following this avenue of research is outside the scope of this paper, but may be examined in future studies.

As the mean age of our control group was 25.2, the method performed better with adults. In two younger patients (age<10) more additional clusters were identified, most likely resulting from differences in myelination and erroneous registration due to more pronounced anatomical (i.e. head and brain size) differences.

As the method proved to be sensitive to a wide range of malformations and even to more pronounced physiological variations, more carefully selected control group(s) of matching age would increase specificity (Fig 9) and better characterization of abnormal tissue microstructure. Nevertheless, since MCDs associated with the epileptic seizures were identified in all but one cases, even with approximately 17 years of age difference, it was established that detecting disrupted tissue microstructure using tensor eigenvalues, based on the Mahalanobis-distance is indeed feasible and may aid in single subject’s radiological evaluation. Additional case studies, not included in the present article, demonstrated, that the effects of large anatomical abnormalities, higher level of subject motion, or differences in scan parameters (even with a robust DWI processing pipeline with thorough motion correction and high performance spatial registration) leads to a more severe artefact contamination in the results, than the effect of age difference.

With clusters observed partially outside the brain parenchyma (typically in the sulci) or evidently following the GM-CSF boundary, regardless of the underlying tissue macrostructure, registration performance may also be a major effect; potentially causing a high number of artefactual clusters, not all of which could be filtered out with the TPM-based cluster-evaluation method. Fortunately, such clusters are easily identifiable as obvious artefacts, and so are the results of possible missing slices, postoperative resection sites, large anatomical variations (e.g. agenesis of the corpus callosum), or large-scale shifts, rotations, or shears.

Papers in the field of automated lesion detection usually examine single types of pathologies, for example patients with FCDs [9, 10, 44, 78], benefiting from the more specific research question. On the other hand, as clinical practice suggests that different types of MCDs tend to develop together, our patient group of individuals with mixed pathologies more faithfully represents typical cases of drug resistant epilepsies [3]. The multidimensional approach proved to be sensitive to the different types of malformations, which is a satisfying result for a potential lesion detection method, however, if the framework is to be extended with data from other modalities in future studies, feature selection analysis would benefit from selecting cases with single types of MCDs.

The distance metric itself cannot shed light on the nature of the altered diffusion profile, potentially resulting from several aforementioned normal, pathological, or compensational processes, which could also be varying across individuals. Therefore the generalization of findings would benefit from group-based measures of the alterations; the identified regions of disrupted microstructure may be subjected to subsequent conventional testing, for example, exploring whether FA is increased or decreased in the region, or assessing abnormal connectivity through tractography by using the clusters as seed regions [79]. In future studies, such subsequent examinations, potentially including group-based measures derived from patients with similar pathologies, could help discerning between direct and compensatory effects, explaining some of the observed distant WM clusters.

The proposed method proved successful in combining separate eigenvalue maps, benefiting from the advantages of the multidimensional approach, and achieved sufficient sensitivity in detecting abnormal diffusion profile. The straightforward application of analytically-derived critical values [54] allowed making strong inferences, although specificity was limited due to registration artefacts and normal or pathological variations: effects inherent to all single subject examinations.

### Limitations

The wide range of pathologies and the technical impediments may constrain the generalization of findings, nevertheless, as the major goal of the present study was to introduce a new method of statistical evaluation, these predicaments may prove useful in assessing the flexibility of the method.

Using study specific templates, e.g. the DARTEL approach in the present study may be considered a limitation, especially when evaluating possible diagnostic tools, nevertheless, the aim of the current paper was to demonstrate the value of the Mahalanobis-distance based approach in single patient vs control comparisons. Further analyses using multi-center multi-scanner data may further warrant the evaluation of the diagnostic potential of a Mahalanobis-distance based lesion detection tool.

During additional patient examinations, not presented in the article, we found that a system upgrade also affected the outcome of the statistical analyses, leading to apparent alterations in almost the entire WM, this effect may also most probably stem from the rather homogeneously collected control data. A multi-center, multi-scanner investigation, like mentioned above, may prove to be useful in overcoming such limitations.

## Conclusions

Taken together, the proposed Mahalanobis-distance based method efficiently combined information from maps of the three diffusion tensor eigenvalues on the voxel-level. Altered diffusion profiles corresponding to malformations of cortical development in single subject vs. control group examinations were detected as outlier values in the voxel-wise multidimensional distributions.

Searching for pathological brain regions of individuals as outliers, using the Mahalanobis-distance in evaluation of diffusion weighted imaging data (even with more sophisticated models for processing, if necessary) seems to be a viable approach, and as the calculations could easily cover data from other modalities, this evaluation method may substantially advance the field of quantitative MRI.

## Supporting information

S1 Fig Examples of the observed clusters in the leave-one-out examinations of healthy control subjects, using critical values corrected for controlling the FWE rate. Cluster masks overlaid on each individual’s T1-weighted image. Typical clusters that remained after the filtering steps, emerged deep in the sulci or close to the GM-CSF boundary (A, B, C, and D) with small sizes (16.21 voxels in average), and also in the WM in some cases (E, F). Axial and coronal slices are presented in neurological orientation, i.e. left side is on the left.
(TIF)

S2 Fig Comparing the location of distant WM clusters to tractography. Deterministic DTI tractography (performed in ExploreDTI) revealed, that several of the distant WM clusters are connected to the primary lesions, for example in a 33 y.o. female patient with multiplex right temporal closed-loop schizencephaly and subependymal heterotopia (left), and in a 27 y.o. male patient with presumed polymicrogyria or FCD in the left inferior frontal gyrus and the posterior third of the left insula (right). Axial and coronal slices presented in neurological orientation, i.e. left side is on the left.
(TIF)

S3 Fig **Coronal 2D FLAIR images, and MAP07 junction maps, raw, and final, clustered D**^**2**^**-images (red) and lesion masks (green) overlaid on T**_**1**_**-weighted images of the remaining cases.** Bilateral frontal WM signal alterations with presumably ischaemic origin -14 y.o. female patient (panel A). Cortical dysgenesis in the right parieto-occipital sulcus -16 y.o. male patient (panel B). Histology confirmed focal gliosis -16 y.o. male patient (panel C). Left temporo-basal DNT and hippocampal sclerosis -15 y.o. female patient (panel D). Multiplex right temporal closed-loop schizencephaly and subependymal heterotopia -33 y.o. female patient (panel E). Focal cortical dysplasia in the left middle frontal gyrus– 7 y.o. male patient (panel F). Presumed PMG or FCD in the basal region of the left inferior frontal gyrus and the posterior pat of the insula (panel G). Right temporal closed-loop schizencephaly and subependymal heterotopia -35 y.o. male patient (panel H). Coronal slices presented in neurological orientation, i.e. left side is on the left, coronal slices of the 2D FLAIR images were angulated perpendicular to the hippocampi.
(TIF)

S1 Table Patient details, description of the separate malformations, and comparative evaluation of the results. Results of independent lesion detection (MAP07) and the proposed Mahalanobis-distance based method were evaluated with the expert neuroradiologist (PB); apart from three cases, the diagnoses of MCD subtypes were based on imaging.
(PDF)
