# Supplementary figures and images for: Personalized microstructural evaluation using a Mahalanobis-distance based outlier detection strategy on epilepsy patients’ DTI data – Theory, simulations and example cases
Source: PLoS One. 2019 Sep 23;14(9):e0222720. doi: 10.1371/journal.pone.0222720 (PMC6756533; doi:10.1371/journal.pone.0222720)

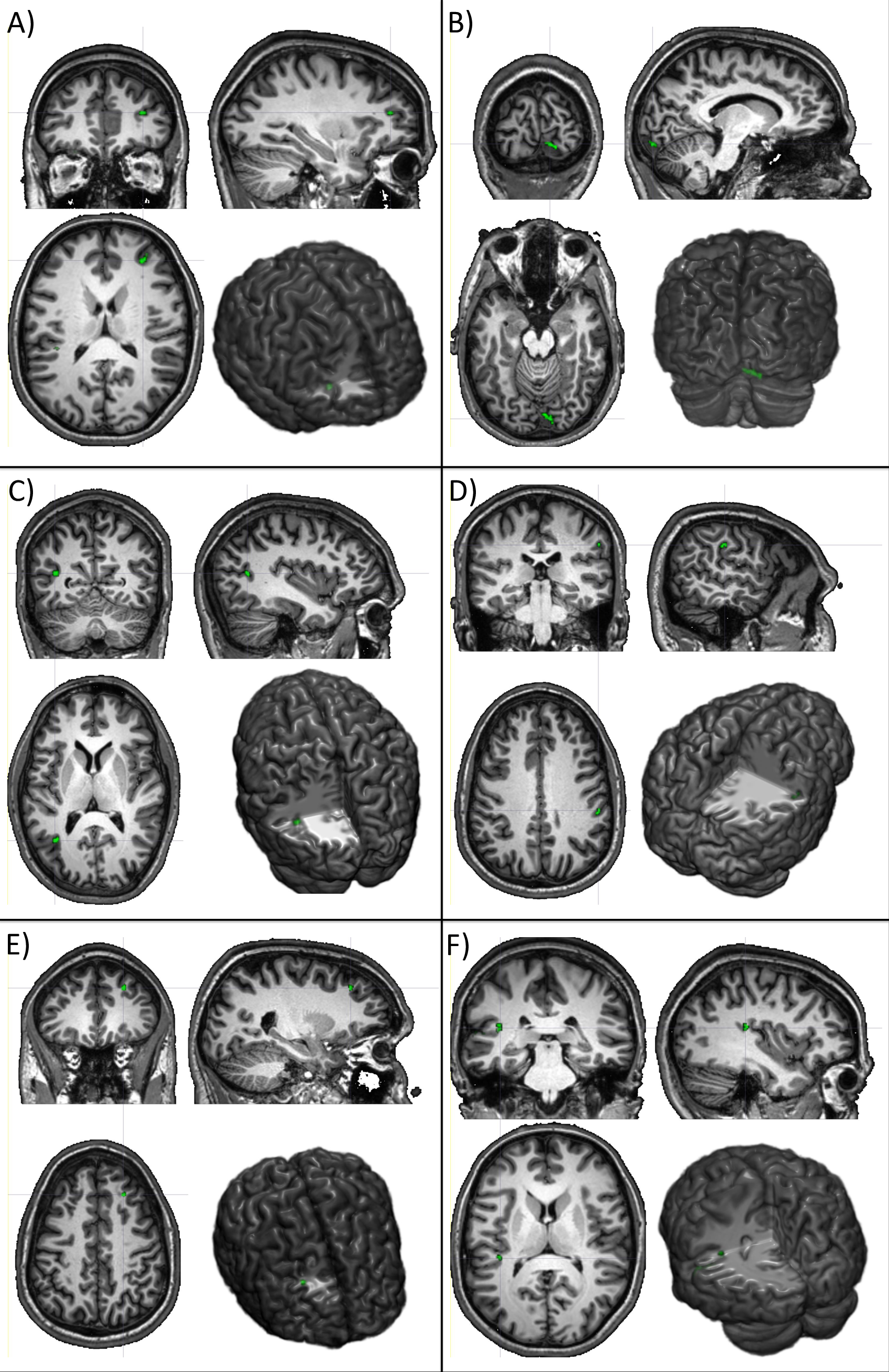

Supplement: S1 Fig — Cluster masks overlaid on each individual’s T1-weighted image. Typical clusters that remained after the filtering steps, emerged deep in the sulci or close to the GM-CSF boundary (A, B, C, and D) with small sizes (16.21 voxels in average), and also in the WM in some cases (E, F). Axial and coronal slices are presented in neurological orientation, i.e. left side is on the left. (TIF) [file pone.0222720.s001.tif]

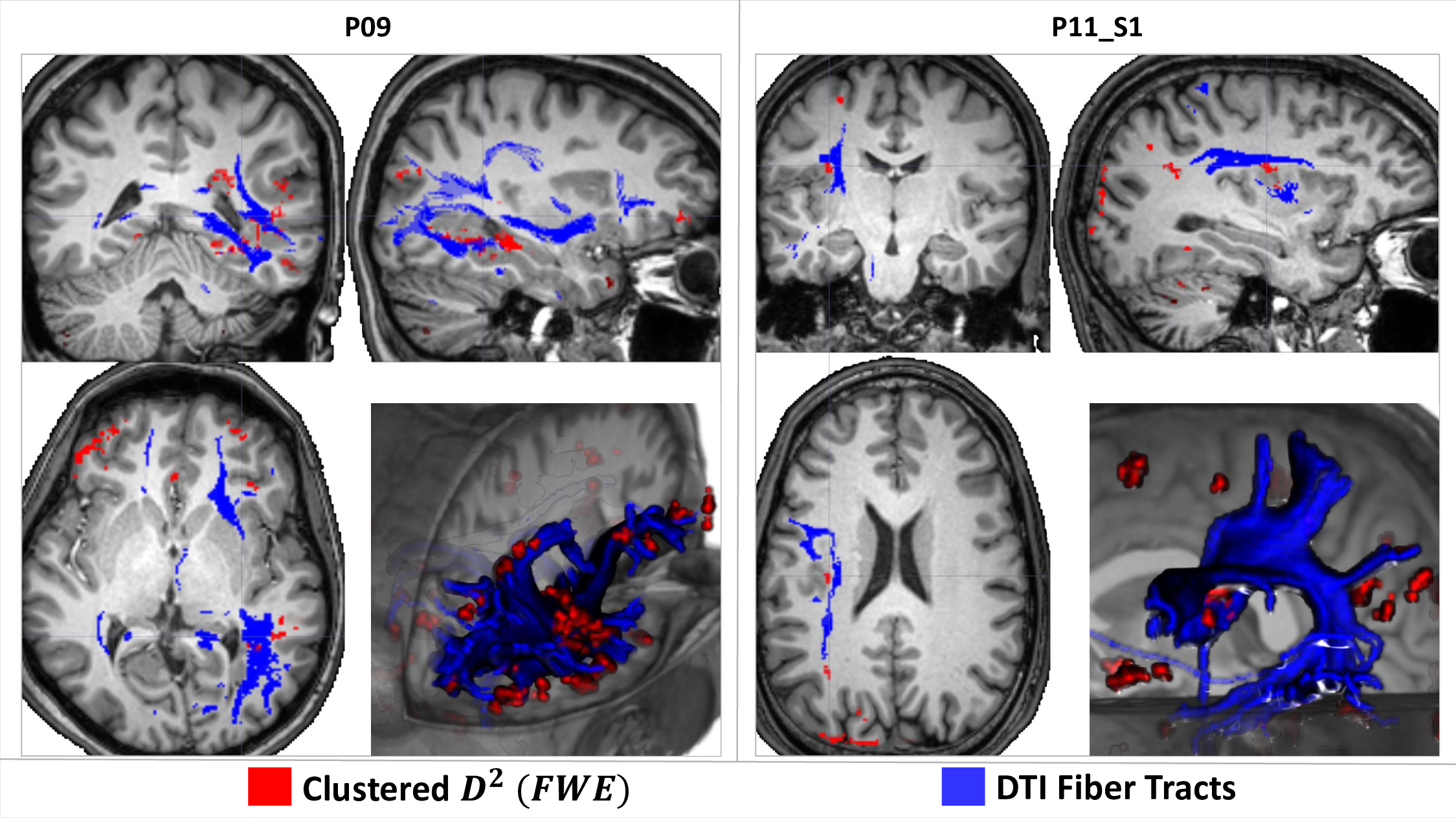

Supplement: S2 Fig — Deterministic DTI tractography (performed in ExploreDTI) revealed, that several of the distant WM clusters are connected to the primary lesions, for example in a 33 y.o. female patient with multiplex right temporal closed-loop schizencephaly and subependymal heterotopia (left), and in a 27 y.o. male patient with presumed polymicrogyria or FCD in the left inferior frontal gyrus and the posterior third of the left insula (right). Axial and coronal slices presented in neurological orientation, i.e. left side is on the left. (TIF) [file pone.0222720.s002.tif]

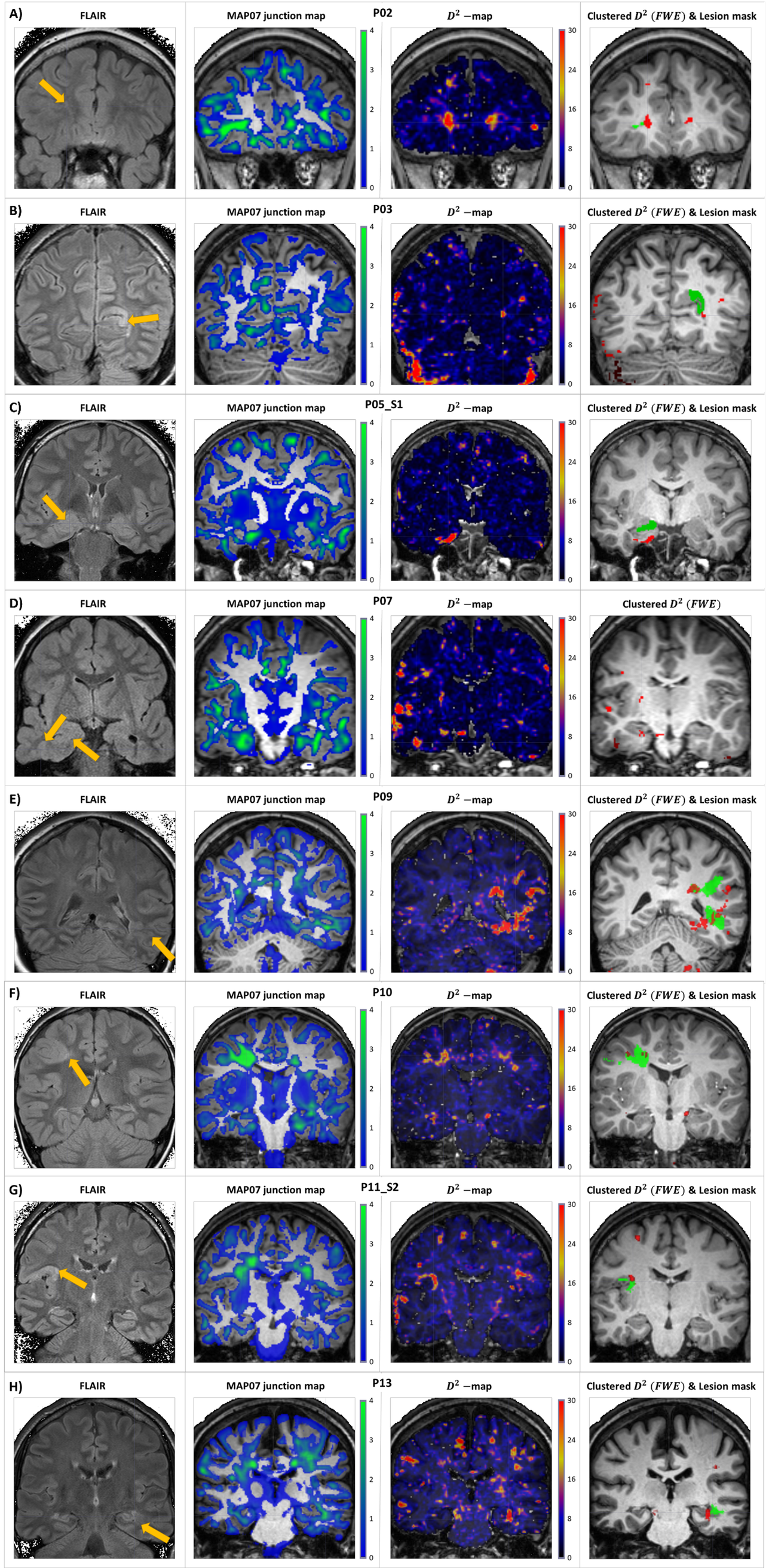

Supplement: S3 Fig — Coronal 2D FLAIR images, and MAP07 junction maps, raw, and final, clustered D2-images (red) and lesion masks (green) overlaid on T1-weighted images of the remaining cases. Bilateral frontal WM signal alterations with presumably ischaemic origin -14 y.o. female patient (panel A). Cortical dysgenesis in the right parieto-occipital sulcus -16 y.o. male patient (panel B). Histology confirmed focal gliosis -16 y.o. male patient (panel C). Left temporo-basal DNT and hippocampal sclerosis -15 y.o. female patient (panel D). Multiplex right temporal closed-loop schizencephaly and subependymal heterotopia -33 y.o. female patient (panel E). Focal cortical dysplasia in the left middle frontal gyrus– 7 y.o. male patient (panel F). Presumed PMG or FCD in the basal region of the left inferior frontal gyrus and the posterior pat of the insula (panel G). Right temporal closed-loop schizencephaly and subependymal heterotopia -35 y.o. male patient (panel H). Coronal slices presented in neurological orientation, i.e. left side is on the left, coronal slices of the 2D FLAIR images were angulated perpendicular to the hippocampi. (TIF) [file pone.0222720.s003.tif]
