# Supplementary material for: Personalized microstructural evaluation using a Mahalanobis-distance based outlier detection strategy on epilepsy patients’ DTI data – Theory, simulations and example cases
Source: PLoS One. 2019 Sep 23;14(9):e0222720. doi: 10.1371/journal.pone.0222720 (PMC6756533; doi:10.1371/journal.pone.0222720)
Supplement: S1 Table — Results of independent lesion detection (MAP07) and the proposed Mahalanobis-distance based method were evaluated with the expert neuroradiologist (PB); apart from three cases, the diagnoses of MCD subtypes were based on imaging. (PDF) [file pone.0222720.s004.pdf]

S1 Table: Patient details, description of the separate malformations, and comparative evaluation of the results

Code	Sex	Age	Location	Neuroradiology assessment	MAP07	Raw D^2 images	Thresholded and clustered D^2
P01	m	14	Right superior temporal gyrus	Presumed FCD or PMG; subsequent histology ruled out tumor or dysgenesis	Positive in a small cluster	Clear positivity with high distance values	Positive
			Right amygdala, uncus, and hippocampus	More voluminous on T_1w, with higher intensity on FLAIR images; presumable hippocampal sclerosis	The surrounding WM is marked positive	High D^2 values in the hippocampus, reaching significance in the medial aspect	Positive on the medial aspect
			Right insula	Thicker, but iso-intense GM on T_1w and FLAIR images, MCD can not be ruled out	Small positive cluster in the vicinity of signal disturbances	Large area of positivity with several medium-sized clusters of significant distance values	Positive
			Fronto-basal regions, occipital and left temporal lobes, and the cerebellum	No sign of abnormalities	Several, pronounced positivities distant to the above-listed lesions	Clusters of probable artefacts; neuroradiologically confirmed MCDs are distinguishable by a halo of voxels with higher yet non-significant D^2 that surrounds the significant regions	Large fronto-basal, cerebellar, and occipital artefacts, with smaller clusters in the left temporal lobe contralateral to the neuroradiologically confirmed pathologies on the right side
P02	f	14	Left and right cella media of the lateral ventricle and the border of the right occipital WM	Small lesions on the ventricular wall consistent with subependymal heterotopia	Negative	High, albeit not significant D^2 values in the WM surrounding the MCDs in the ventricular walls	The paraventricular MCDs are positive with clusters smaller than the lesions
			Bilateral frontal WM	Bilateral frontal WM signal alterations with presumably ischemic origin	Positive	Large positivities in the frontal WM and in the corpus callosum	Large positive clusters consistent with the bilateral frontal ischemic WM-lesions and a cluster in the corpus callosum
			Terminal WM	Possibly due to incomplete myelination (age difference between patient and controls)	Positive	High D^2 values in the terminal WM, significant regions surrounded by non significant voxels	Several medium-sized positive clusters in the terminal WM

S1 Table 1 (Continued)

<table>
<tr><th>Code</th><th>Sex</th><th>Age</th><th>Location</th><th>Neuroradiology assessment</th><th>MAP07</th><th>Raw D^2 images</th><th>Thresholded and clustered D^2</th></tr>
<tr><td rowspan="2">P03</td><td rowspan="2">m</td><td rowspan="2">16</td><td>Right parieto-occipital sulcus</td><td>Stable FLAIR signal intensity alterations on multiple follow-ups in the right parieto-occipital sulcus consistent with cortical dysgenesis</td><td>Negative</td><td>Positive voxels clustered around the neuroradiologically confirmed MCD, a halo of voxels with higher yet non-significant D^2 surrounds the significant regions</td><td>The location of the MCD is pointed out by small clusters of significant D^2 in the adjacent WM</td></tr>
<tr><td>Right occipital and bilateral frontal regions, right superior temporal WM</td><td>No sign of abnormalities</td><td>Several positive clusters</td><td>Clusters with high D^2 values in the right occipital, and bilateral frontal regions representing obvious registration artefacts; a positive region in the right superior temporal WM contralateral to the neuroradiologically confirmed lesion; clusters with higher D^2 values in the genu and splenium of the corpus callosum</td><td>There are significant voxels in the regions observed on the raw D^2 map in a distribution consistent with registration artifacts</td></tr>
<tr><td>P04</td><td>f</td><td>17</td><td>Right temporo-occipital region</td><td>Clearly visualized FCD, (presumably type IIB) on T_1w and FLAIR images</td><td>Positive</td><td>Region of voxels with high, but non-significant D^2 values around the MCD, with several voxels above the FDR-corrected threshold of significance, but only two voxels surviving FWE correction</td><td>Negative with FWE, but positive with FDR-correction</td></tr>
<tr><td>P05_S1</td><td rowspan="3">m</td><td>16</td><td rowspan="3">Left amygdala, uncus, and hippocampus</td><td rowspan="3">Histology confirmed focal gliosis. The increasing involvement of the contralateral structures in the subsequent examinations (2, and 6 years later) may be resulting from damaged WM caused by the seizures originating from the left hemisphere, propagating through the interconnecting fibers.</td><td>Positive</td><td rowspan="3">Raw D^2 values increased with time demonstrating expanding involvement of the left and right amygdala, uncus and hippocampus.</td><td rowspan="3">Clear positive results, subsequent examinations showed contralateral regions of abnormal diffusion; results of the last examination seemed to be more localized, with smaller clusters than previously</td></tr>
<tr><td>P05_S2</td><td>18</td><td>Positive, right side shows higher D^2 values than on previous examinations</td></tr>
<tr><td>P05_S3</td><td>22</td><td>Positive in the left focal gliosis, but there are positive clusters in the contralateral side</td></tr>
</table>

S1 Table 1 (Continued)

Code	Sex	Age	Location	Neuroradiology assessment	MAP07	Raw D^2 images	Thresholded and clustered D^2
P06	f	8	Right medio-frontal part of the cingulate gyrus	Apparent Cortical dysplasia and mild WM glial surplus; bilateral frontal-medial cortical disgenesis with right hemisphere predominance, presumably polymicrogyria	The neuroradiologically confirmed lesion is clearly positive alongside several other clusters including fronto-basal areas and the genu of corpus callosum	Most of the neuroradiologically confirmed lesion site clearly shows high D^2 values just below the threshold of significance (D^2≈20), there are several clusters with high D^2 including fronto-basal areas and the genu of corpus callosum	Only the posterior right frontal-cingular part of the neuroradiologically confirmed lesion is significant; there are several small clusters in the bilateral frontal WM and the genu of the corpus callosum
P07	f	15	Left temporo-basal	Confirmed DNT in the left temporo-basal region. A subsequent lesionectomy was incomplete and did not affect the hippocampus, seizure-freedom was not achieved	Widespread positive clusters throughout the brain, with the left hippocampus and left Heschl's gyrus also marked positive	Large clusters of high D^2-values in the left temporo-basal region and the left temporal pole, most pronounced around the neuroradiologically confirmed lesions	Clear positive result in the left temporo-basal areas, with several positive clusters in the left temporal lobe
			Left hippocampus	Apparent hippocampal sclerosis, dysgenesis cannot be ruled out			The left hippocampus is evidently marked
			Heschl's gyrus	Questionable signal alteration that proved to be negative on subsequent examinations		Left superior temporal gyrus and the Heschl's gyrus are marked by high D^2 values	One significant cluster is observed in the left Heschl's gyrus
P08	m	46	Left amygdala and hippocampus	Dysgenesis, and partial hippocampal sclerosis	Surrounding tissue around the left hippocampus is marked positive, along with most of the temporal and occipital WM-GM boundary, the right cingulum, the right occipital WM, and the left occipital pole	Both hippocampi (with evident left predominance) are positive	The head of the left hippocampus and a smaller part of the right is positive
			Posterior-superior part of the Sylvian fissure	Most likely pulsation artefact resulting from anatomical variation of arteries in the vicinity		Small cluster is observed with high D^2 values	One small cluster of high D^2 values above the threshold of significance
			Occipital WM	No clear sign of abnormalities		Several regions of high D^2-values	Several small-medium sized clusters in the occipital WM

S1 Table 1 (Continued)

Code	Sex	Age	Location	Neuroradiology assessment	MAP07	Raw D^2 images	Thresholded and clustered D^2
P09	f	33	Right temporal lobe	Multiplex right temporal closed-loop schizencephaly and subependymal heterotopia, the latter also present in the peritrigonal region, connected to the cortex of the Sylvian fissure. Postictal, or dysgenetic changes in the right amygdala and hippocampus	Most of the right temporal lobe is positive, along with parts of the occipital and frontal areas	Large areas of the right temporal and occipital lobes and clusters in both frontal lobes are positive with high D^2 values	Large positive clusters in and around the neuradiologically confirmed lesions in the right temporal lobe. The connection between the frontal and occipital clusters and the primary lesions are verified by DTI-tractography
P10	m	7	Left middle frontal gyrus	Presumed focal cortical dysplasia in the left middle frontal gyrus	The left medial frontal gyrus is clearly positive	A larger cluster of high D^2 values around the neuroradiologically confirmed lesion and contralateral to it (the latter below the level of significance).	The neuroradiologically confirmed lesion in the left medial frontal gyrus is clearly positive
			Left hippocampus	Malrotation of the left hippocampus	Large areas positive in the temporal lobes bilaterally	High D^2 values in the temporal lobes bilaterally, with some significant voxels in the left hippocampus	The left hippocampus is positive
			Cingulum, terminal and occipital WM	Possibly due to incomplete myelination (age difference between patient and controls)	Large areas positive in the cingulum, and the terminal WM with smaller clusters at the occipital GM-WM boundary	Small clusters with few voxels above the level of significance and several with medium-high D^2 values	Two significant clusters in the left cingulum, and few smaller clusters in the terminal and occipital WM

S1 Table 1 (Continued)

Code	Sex	Age	Location	Neuroradiology assessment	MAP07	Raw D^2 images	Thresholded and clustered D^2
P11_S1	m	27	Basal region of the left inferior frontal gyrus and the posterior third of the left insula	Malformation of cortical development, presumably polymicrogyria or FCD	Positive	Medium-sized area with high D^2 values in and around the neuroradiologically confirmed lesion	Positive
			Left frontal, bilateral occipital and temporal WM	No clear sign of abnormalities	Positivities in parts of the left frontal, and bilateral temporal and occipital lobes and in most of the corona radiata bilaterally	Several high D^2 clusters, with some voxels above the significance threshold	Clusters in the left frontal, the bilateral occipital, and the left terminal WM, the latter is evidently connected to the primary lesion, verified by DTI tractography. Occipital registration artefacts are easy to identify by their configuration
P11_S2	m	27	Basal region of the left inferior frontal gyrus and the posterior third of the left insula	Malformation of cortical development, presumably polymicrogyria or FCD	Positive	Medium-sized area with high D^2 values in and around the neuroradiologically confirmed lesion	Positive
			Left frontal, bilateral occipital and temporal WM	No clear sign of abnormalities	Positivities in parts of the left frontal, and bilateral temporal and occipital lobes and in most of the corona radiata bilaterally	Several high D^2 clusters, with some voxels above the significance threshold. The right insula, contralateral to the neuroradiologically confirmed lesion contains higher D^2 values in a larger volume than previously	Most WM clusters further apart from the neuroradiologically confirmed lesion are still present. Fewer artefacts than previously

S1 Table 1 (Continued)

Code	Sex	Age	Location	Neuroradiology assessment	MAP07	Raw D^2 images	Thresholded and clustered D^2
P12	m	14	Left hippocampus	Left hippocampal sclerosis	Negative	Significantly high D^2 values in the left hippocampus, with high values in the surrounding WM	Positive
			Left temporal pole	Malformation of cortical development (FCD-IIIa) in the left temporal pole	Most of the left temporal lobe is marked positive	High D^2 values in the WM of the left temporal pole, most voxels below the threshold of significance	One small positive cluster in the WM close to the left temporal pole
			Right temporal pole	Gliotic cyst with approx. 2mm size in the right temporal pole	Most of the right temporal lobe is marked positive	Significantly high D^2 values in the volume of the cyst, high D^2 values in the surrounding tissue	Positive
			WM in the posterior part of the left and right superior frontal gyri	WM FLAIR signal alterations in the right superior frontal gyrus	Clusters in the superior corona radiata are marked positive bilaterally	High D^2 values in the superior frontal WM of both hemispheres with right predominance	One cluster in either hemispheres in the posterior parts of the superior frontal gyri
P13	m	35	Right temporal lobe	Right temporal closed-loop schizencephaly and subependymal heterotopia	Most of the temporal lobes and the superior corona radiata are marked positive bilaterally	The right temporal lobe contains the most voxels with high D^2-values clearly centered around the neuroradiologically confirmed lesion	Large significant clusters in the right temporal lobe and the hippocampus clearly mark the pathology
			Right occipital pole	Several WM signal alterations in the occipital poles bilaterally with right predominance. May be due to circulatory disturbance(s) during the 2nd trimester (the same cause may be behind the schizencephaly, as well).	Positive, but with smaller clusters and weaker effect size	Frontal and occipital lobes contain regions of high D^2 values bilaterally with right predominance	Several small clusters in the occipital lobes bilaterally with right predominance
			Frontal lobes bilaterally and the anterior part of the right internal capsule	No clear sign of abnormalities	The frontal WM is marked positive bilaterally	Small clusters of higher D^2-values with a few voxels above the level of significance	Few clusters in the frontal lobes and one in the anterior part of the right internal capsule

Results of independent lesion detection (MAP07) and the proposed Mahalanobis-distance based method were evaluated with the expert neuroradiologist (PB); apart from three cases, the diagnoses of MCD subtypes were based on imaging.
